# Cytokine-chemokine network driven metastasis in esophageal cancer; promising avenue for targeted therapy

**DOI:** 10.1186/s12943-020-01294-3

**Published:** 2021-01-04

**Authors:** Ajaz A. Bhat, Sabah Nisar, Selma Maacha, Tatiana Correa Carneiro-Lobo, Sabah Akhtar, Kodappully Sivaraman Siveen, Nissar A. Wani, Arshi Rizwan, Puneet Bagga, Mayank Singh, Ravinder Reddy, Shahab Uddin, Jean-Charles Grivel, Gyan Chand, Michael P. Frenneaux, Mushtaq A. Siddiqi, Davide Bedognetti, Wael El-Rifai, Muzafar A. Macha, Mohammad Haris

**Affiliations:** 1 Functional and Molecular Imaging Laboratory, Cancer Research Department, Sidra Medicine, Doha, Qatar; 2 Research Department, Sidra Medicine, Doha, Qatar; 3 Laboratory of Cancer Immunogenomics, Cancer Research Department, Sidra Medicine, Doha, Qatar; 4 Translational Research Institute, Academic Health System, Hamad Medical Corporation, Doha, Qatar; 5 Department of Biotechnology, Central University of Kashmir, Ganderbal, Jammu and Kashmir India; 6 Department of Nephrology, All India Institute of Medical Sciences (AIIMS), New Delhi, India; 7 Diagnostic Imaging, St Jude Children’s Research Hospital, Memphis, TN USA; 8 Dr. B. R. Ambedkar Institute Rotary Cancer Hospital (BRAIRCH), AIIMS, New Delhi, India; 9 Department of Radiology, Perelman School of Medicine at the University of Pennsylvania, Philadelphia, USA; 10 Department of Endocrine Surgery, Sanjay Gandhi Post Graduate Institute of Medical Sciences, Lucknow, India; 11 Academic Health System, Hamad Medical Corporation, Doha, Qatar; 12 Watson–Crick Centre for Molecular Medicine, Islamic University of Science and Technology, Awantipora, Jammu & Kashmir India; 13 Department of Internal Medicine and Medical Specialties, University of Genoa, Genoa, Italy; 14 College of Health and Life Sciences, Hamad Bin Khalifa University, Doha, Qatar; 15 Department of Surgery, University of Miami Miller School of Medicine, Miami, Florida USA; 16 Laboratory Animal Research Center, Qatar University, Doha, Qatar

**Keywords:** Esophageal cancer, Cytokines, Chemokines, Inflammation, Tumor microenvironment, Epithelial-Mesenchymal transition, Drug targets, Immune evasion

## Abstract

Esophageal cancer (EC) is a disease often marked by aggressive growth and poor prognosis. Lack of targeted therapies, resistance to chemoradiation therapy, and distant metastases among patients with advanced disease account for the high mortality rate. The tumor microenvironment (TME) contains several cell types, including fibroblasts, immune cells, adipocytes, stromal proteins, and growth factors, which play a significant role in supporting the growth and aggressive behavior of cancer cells. The complex and dynamic interactions of the secreted cytokines, chemokines, growth factors, and their receptors mediate chronic inflammation and immunosuppressive TME favoring tumor progression, metastasis, and decreased response to therapy. The molecular changes in the TME are used as biological markers for diagnosis, prognosis, and response to treatment in patients. This review highlighted the novel insights into the understanding and functional impact of deregulated cytokines and chemokines in imparting aggressive EC, stressing the nature and therapeutic consequences of the cytokine-chemokine network. We also discuss cytokine-chemokine oncogenic potential by contributing to the Epithelial-Mesenchymal Transition (EMT), angiogenesis, immunosuppression, metastatic niche, and therapeutic resistance development. In addition, it discusses the wide range of changes and intracellular signaling pathways that occur in the TME. Overall, this is a relatively unexplored field that could provide crucial insights into tumor immunology and encourage the effective application of modulatory cytokine-chemokine therapy to EC.

## Background

The incidence of Esophageal cancer (EC) is increasing markedly, and it now represents the 8th most common cancer type and the 6th leading cause of cancer-related deaths worldwide [1], with an estimated 5-year survival rate to be only 15–20% [1]. Esophageal squamous cell carcinoma (ESCC) and esophageal adenocarcinoma (EAC) are the two histological subtypes of EC, which differ in their location, histology, and pathogenesis. While the ESCC subtype represents 90% of all ECs and is more prevalent in developing countries, the incidence of EAC in developed countries is increasing fast [2]. The late clinical presentation of ESCC is associated with locally advanced or distant metastases and is the main reason for poor prognosis [3–5]. In addition, inherent resistance to chemoradiation therapy (CRT) due to tumor heterogeneity and the development of acquired resistance results in treatment failure and low patient survival. Despite significant advances in the treatment strategies, including surgery and chemotherapy and/or radiotherapy [6, 7], the molecular signatures of inherent and acquired resistance account for the prevalent poor prognosis and lack of improved outcomes for several decades.

While cigarette smoking and alcohol consumption are the major risk factors for ESCC, Barrett’s esophagus (BE) and chronic inflammation promote EAC [8]. BE, the main risk factor for EAC, is a premalignant condition in which columnar metaplasia replaces distal esophagus stratified squamous epithelium [9]. BE develops as a result of chronic gastroesophageal reflux disease (GERD), in which acid and bile salts reflux from the stomach into the lower esophagus [10, 11]. Chronic GERD induces high levels of reactive oxygen species (ROS) and oxidative stress in esophageal epithelial cells, the main driving forces for DNA damage and carcinogenesis in EAC. Previous studies have shown that short exposure to bile acids and low pH causes oxidative stress and DNA damage in the esophageal tissues and cells [12, 13]. Besides, acidic bile salt treatment in esophageal cells increases ROS levels, resulting in increased oxidative DNA damage and double-strand DNA breaks [14, 15]. We have shown earlier that APE1 promotes EAC cells’ survival in response to acidic bile salts by enhancing repair of DNA damage and attenuating the JNK-and p38-mediated apoptotic stress response [16]. These findings suggest that APE1 could provide the EAC cells with a survival advantage in response to oxidative stress induced by acidic bile salts. Apurinic / apyrimidinic endonuclease-1 (APE1)/redox effector factor-1 (REF-1) is a multifunctional protein that plays an essential role in the basic excision repair (BER) pathway, critical for the repair of oxidative DNA base damage and the redox-dependent regulation of several transcription factors such as NF - kB, p53, AP-1, HIF-1α and EGR-1 [17, 18].

Recently our group has also shown that exposure of BE and EAC cells to acidic bile salts, under conditions that closely mimic GERD, activates EGFR and STAT3 signaling in an APE1 redox-dependent manner. We noted a significant increase in mRNA expression of IL-6 and IL-17A after exposure to bile salts [19]. In another interrelated study, we showed upregulation of the matrix metalloproteinase MMP-14, which in turn activated MMP-2, leading to the degradation of the extracellular matrix (ECM) and increased invasion of EAC cells [20].

Recent studies have conclusively established an essential role of the tumor microenvironment (TME) in EC progression and metastasis. It has been demonstrated that EC TME is enriched for pro-inflammatory cytokines, chemokines, and growth factors [21–23], and their complex cross-talk with their receptors influences the development and progression of EC. A comprehensive understanding of the complex inter-connected stromal networks via these secretory factors offers an opportunity to identify novel targets with diagnostic, prognostic, and therapeutic potential. This review focuses on the current understanding and functional impact of deregulated cytokines and chemokines in EC. Their practical importance is in imparting an aggressive EC phenotype by contributing to Epithelial-Mesenchymal Transition (EMT), angiogenesis, immunosuppression, metastatic niches, and therapeutic resistance. We also discussed the wide range of changes and intracellular signaling pathways occurring in the TME that could be exploited in EC as a future therapeutic strategy.

## Esophageal cancer microenvironment

Esophageal cancer TME is a very dynamic and complex ecosystem entailing cellular components (cancer-associated fibroblasts (CAFs), immune cells, endothelial cells, pericytes, adipocytes), extracellular matrix proteins (collagen, elastin fibers, fibronectins, proteoglycans, hyaluronic acid, osteopontin, periostin, and SPARC (secreted protein acidic and rich in cysteine, also known as osteonectin or BM-40) and secretory proteins including cytokines, chemokines and many growth factors secreted by tumor and stromal cells. The TME is also infiltrated by immunosuppressive cells, such as regulatory T (Treg) cells, myeloid-derived suppressor cells (MDSCs), and tumor-associated macrophages (TAMs) [24, 25].. Cancer cells are known to secrete several soluble factors, including cytokines, chemokines, and growth factors, which help the immune cells reprogram the surrounding microenvironment to promote tumor growth, metastasis, and resistance to CRT. In advanced tumors, various chemokines and pro-inflammatory cytokines that promote cancer are abundant, whereas cytokines that inhibit tumor growth are usually lacking [26–28]. Durand RE in his keynote address at the “Third International Conference on The Interaction of Radiation Therapy and Systemic Therapy, Asilomar Conference Center, Monterey, CA, 9-12 March 1990” described the importance of the TME in modifying the CRT response [29], and proposed that the TME components should be exploited for targeted therapy. Since then, extensive studies have conclusively established the importance of TME in promoting CRT resistance through the activation of multiple tumor-associated signaling pathways [30–33]. TME stromal cells secrete cytokines, chemokines [34], and exosomes [35] to alter cancer cell signaling and metabolic pathways [36, 37]. Also, stromal cells, particularly CAFs, regulate angiogenesis [38] and change the composition and biophysical features of the extracellular matrix [39], hindering the delivery of therapeutics to the cancer cells. Together, these factors confer CRT resistance.

As in most tumors, EC TME is immuno-suppressive, favoring tumor growth and the development of an aggressive phenotype. The types of immune cells associated with TME are mostly associated with cytokine production, broadly classified as anti-tumorigenic (IL-12, IFN-γ, TRAIL), pro-tumorigenic (IL-6, IL-23, IL-10, IL-17), and cytokines with direct roles on cancer cells’ signaling (TGF-β, TNFα, IL-6, FasL). The MDSCs are a heterogeneous group of immature myelocytes that can suppress both innate and antigen-specific immune response that has been extensively studied in EC pathogenesis. Though the involvement of TAMs has not been extensively studied in EC, their infiltration into the TME by Monocyte chemoattractant protein-1 (MCP-1/CCL2) (secreted by cancer cells) and production of pro-angiogenic factors are well known [40, 41]. Furthermore, the presence of TAMs is associated with inadequate therapeutic response and poor EC patient prognosis [42]. The other immunosuppressive T cells, such as Tregs and Th17, are recruited by tumor cell-derived chemokines CCL17 and CCL22 and are known to promote EC pathogenesis. In particular, the presence of increased Tregs in the EC TME is associated with invasion, metastases, disease severity, decreased survival, and thus considered of prognostic significance [43–46]. Though the role of Th17 cells in cancer is still a subject of debate [47, 48], an increased presence of Th17 cells in both peripheral blood and tumor EC tissues correlates with advanced disease stage [49, 50]. Like DCs, Tregs and Th17 cells are heterogeneous and have several context-dependent functions, forcing a deeper understanding of their role in EC as future therapeutic targets.

The cytokines and chemokines secreted by the immune cells mediate cancer-stromal interactions and activate several downstream effector pathways such as JAK/STAT, NF-κβ, NOTCH to mediate various properties of cancer hallmarks [51]. Activation of STAT3 by its major inducers, IL-6, and IL-6Rα, has been associated with poor prognosis in patients undergoing esophagectomy [52]. The STAT3 inhibitor Stattic has been shown to increase radiosensitivity in EC in preclinical models [53]. Several small-molecule STAT3 inhibitors have shown potential in the preclinical setting [54, 55]. However, none of these are in the clinical trial phase yet, asking for further robust research in this field. Overexpression and activation of the p65 subunit of NF-κB have been reported in EC specimens, and its association with the resistance to 5-fluorouracil is demonstrated in cultured EC cell lines [56]. IL-8, which is one of the foremost upstream regulators of NF-κB signaling, has been associated with inflammation, disease progression, metastases, and poor prognosis in EC patients [57]. The CC chemokine, CCL5 (also known as RANTES), is a downstream target of the NF-κB pathway [58] known to promote tumor cell proliferation, invasion, and angiogenesis [58] by facilitating the recruitment of eosinophils, monocytes, T cells, and basophils [59, 60]. A recent study found that the treatment of EC cells with radiotherapy increases the levels of Dioxygenase 12-lipoxygenase (12-LOX), modulating CCL5 expression through the AKT/NF-κB pathway [61]. 12-LOX is an enzyme that metabolizes arachidonic acid into 12-hydroxyeicosatetraenoic acid and contributes to the macrophages’ polarity towards the M2 subtype. 12-LOX over-expression was observed to increase the levels of CCL5, which in turn helped to recruit and repolarize M2 macrophages [61]. Increased 12-LOX enzyme levels are associated with multiple cancer types where it contributes to tumor progression and metastasis [62–64]. The cross-talk between STAT3 and NF-κβ signaling pathways is well known during the inflammatory process in EC tumorigenesis [65] and the role in promoting resistance to CT [66].

## Cytokine/chemokine expression in esophageal cancer

**Fig. 1:**
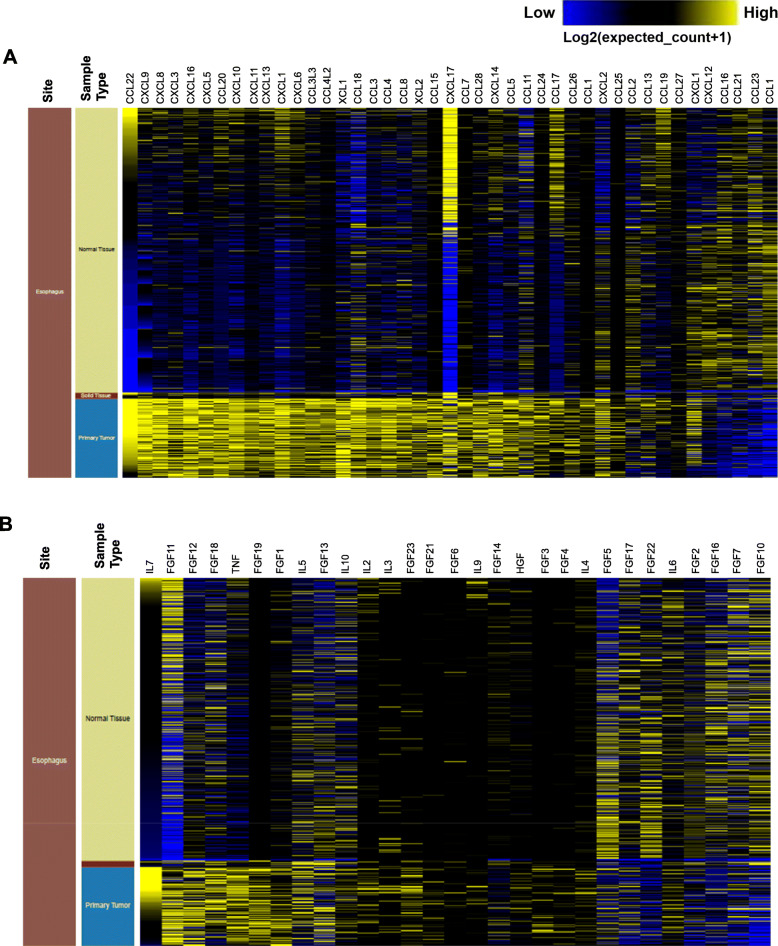
Cytokine/chemokine profile in esophageal tumor and normal tissues. Heat map showing cytokine/chemokine gene expression across normalized GTEX (Normal Tissue, *n* = 8000) and TCGA- ESCA (Tumor tissue, *n* = 10,431) data sets analyzed by using UCSC Xena (https://xenabrowser.net/datapages/)

EC pathogenesis is associated with the deregulation of many cytokines and chemokines (Fig. 1a and b). Cytokines act as markers of metastasis and angiogenesis, the root cause of cancer mortality [67]. While most cytokines promote cancer proliferation, there are other cytokines such as IFN-γ and the interleukins such as IL-27, IL-23, IL-12, and IL-2 that function as tumor suppressors by stimulating antitumor immune responses [68], and cytokines, including IL-6, IL-8, and IL-1β, are associated with transcriptional regulation [69]. Pro-inflammatory cytokines, including interleukin-1 (IL-1) [70], interleukin-6 (IL-6) [71], and tumor necrosis factor-α (TNF-α) [72] have been linked with EC pathogenesis. IL-1 is involved in promoting angiogenesis through the production of vascular endothelial growth factor (VEGF) [73]. The downregulated expression of IL-1 receptor antagonist (IL-1RA) in EC is associated with tumor progression and poor survival [74]. Overexpression of IL-1RA in IL-1α expressing KYSE410 EC cells decreased proliferation and reduced expression of VEGF-A [74]. On the contrary, no effect of IL-1RA overexpression was observed in EC9706 cells (low IL-1α expression) proliferation and VEGF-A expression [74]. IL-1β is a pro-inflammatory cytokine which is highly expressed in EC tissues and promotes invasion and migration [75]. Overexpression of IL-1β has been reported in EC and is involved in increased cell proliferation and associated with poor EC patient prognosis [70]. Another stromal cell-derived cytokine IL-11 is highly expressed in EC, and its knockdown inhibits EC cell proliferation (Eca109 and KYSE410) [76]. Similarly, overexpression of IL-33 has been reported in EC patients and its knockdown reduced the invasive and metastatic potential in KYSE-450 and Eca-109 cells [77]. It has been shown that IL-33 promotes tumor invasion by regulating chemokine CCL2 and by recruiting Treg_s_ [77]. IL-19, a member of the IL-10 cytokine family, is highly upregulated in 60% (36 out of 60) of carcinoma tissues from ESCC patients. In contrast, normal esophageal tissue has weak staining or no staining at all, and the expression correlates with advanced stages of the disease and (lymph node and distant) metastasis. In vitro studies have shown that the human EC cell line, CE81T expresses IL-19 as well as its receptor (Il-20R1/R2), and IL-19 can induce cancer cell proliferation, colony formation, and migration, which can be inhibited by anti-IL-19 antibody and anti-IL-20R1 antibody. IL-19 signaling was found to activate P-38, c-Jun N-terminal kinase, ERK1/2, protein kinase B and NF-kB. These factors mediate activation of TGF-β, cyclin B1, matrix metalloproteinase-1 and CXCR4, all related to the proliferation and metastasis of cancer cells. In vivo studies have shown that anti-IL-19 antibody can significantly inhibit the growth of EC [78].

Chemokines and their receptors also play an essential role in tumor initiation and growth [79]. Chemokines have a molecular mass between 8 and 10 kDa, are structurally classified into four groups, such as CXC, CX3C, CC, and C, based on the spacing between the conserved cysteine residues, which are essential for their three-dimensional structure [79, 80]. They are divided into two functional groups, that is, homeostatic and inflammatory. Homeostatic chemokines are produced in specific tissues, for example, CXCL12, CXCL13, CCL14, CCL19, CCL20, CCL21, CCL25, and CCL27 [81], while inflammatory chemokines are produced in response to a pro-inflammatory stimulus, for example, CCL2, CCL3, CCL4, CCL5, CCL11, CXCL8 and CXCL10 [82]. CXC chemokines play a critical role in angiogenesis [83]. Certain chemokines are tumorigenic, and others are tumor suppressors depending on the type of cell or receptor involved [84]. High CX3CL1, CXCL12, and CCL20 expression were reported in serum samples and associated with EC pathogenesis [23]. The expression of CXCL12 and its receptors is associated with tumor invasion, angiogenesis, and lymph node metastasis in EC [85]. The serum concentrations of CXCL12 were higher, whereas, in EC patients, the serum concentrations of its receptor CXCR4 were lower than in healthy controls [85]. Increased expression of the CXCR4 receptor (CXCR4R) was recorded in both the immune and epithelial cells of the IL-1β transgenic mouse model of BE and EAC [86]. Furthermore, the expression of CXCR7, the receptor for both CXCL12 and CXCL11, is overexpressed in both primary tumor lesions and metastatic lymph nodes and is associated with poor EC prognosis [87]. Using both in vitro and in vivo models, knockdown or silencing of CXCR7 was shown to reduce the cell viability and growth of EC [88]. CXCR1 and CXCR2 are both CXCL8 receptors, also known as IL-8 receptors [89]. The serum concentrations of both CXCL8 and CXCR2 were found to be elevated in EC patients, suggesting the significance of CXCL8 as a potential diagnostic tumor marker in EC [90]. Additionally, the CXCL8/CXCR2 axis promotes angiogenesis, tumor development and is associated with poor prognosis [90]. Small inducible chemokine CXCL10 (ligand for CXCR3) is expressed on B cells, T cells, and NK cells. Increased CXCL10 expression correlates with the expression of CD8^+^ T cell markers (CD8 and Granzyme B) in tumor tissues and improved patient survival in ESCC [91, 92].

Chemokine CXCL1 secreted from CAFs has been shown to impart radiotherapy tolerance in ESCC by inhibiting superoxide dismutase 1, an enzyme responsible for ROS scavenging thus increasing ROS accumulation leading to increased DNA damage following radiation [93]. Increased expression of CC chemokines CCL3 (ligand for CCR1, CCR4, and CCR5), CCL4 (ligand for CCR5 receptor) [94], and CCL5 (ligand for CCR1 and CCR5) [95] have been implicated in cancer pathogenesis. Increased CCL3 expression was reported in the serum of EC patients [23]. While, CCL5 was found to be elevated in a cell line derived from metastatic lymph node (TWES-4LN), as compared with a cell line derived from the primary lesion (TWES-4PT), suggesting its role in lymph node metastasis in ESCC [95]. CCL5 knockdown reduced tumor migration and invasiveness in both the cell lines, and therefore targeting the CCL5/CCR5 axis might act as a novel therapeutic strategy for EC [95]. Another CC chemokine, CCL20, and its receptor CCR6 have been implicated in many cancers. Overexpression of CCR6 is associated with proliferation, invasion, and EMT in ESCC [96], while CCL20 overexpression showed a positive correlation with Treg markers (FoxP3 and IL-10) and demonstrated poor patient survival [97]. CCR7, a receptor for ligands CCL19 and CCL21, expressed on mature dendritic and T cells, induces homing of these cells to the lymph node by binding to ligands CCL19 and CCL21 [97]. Higher expression of CCR7 is associated with recurrence, lymph node metastasis, and poor patient survival in ESCC [97–99]. Thus, comprehensive analysis and investigation of the mechanisms underlying cytokine-chemokine network mediated EC pathogenesis could help develop novel therapies.

## Role of cytokine/chemokine network in epithelial-mesenchymal transition

**Fig. 2:**
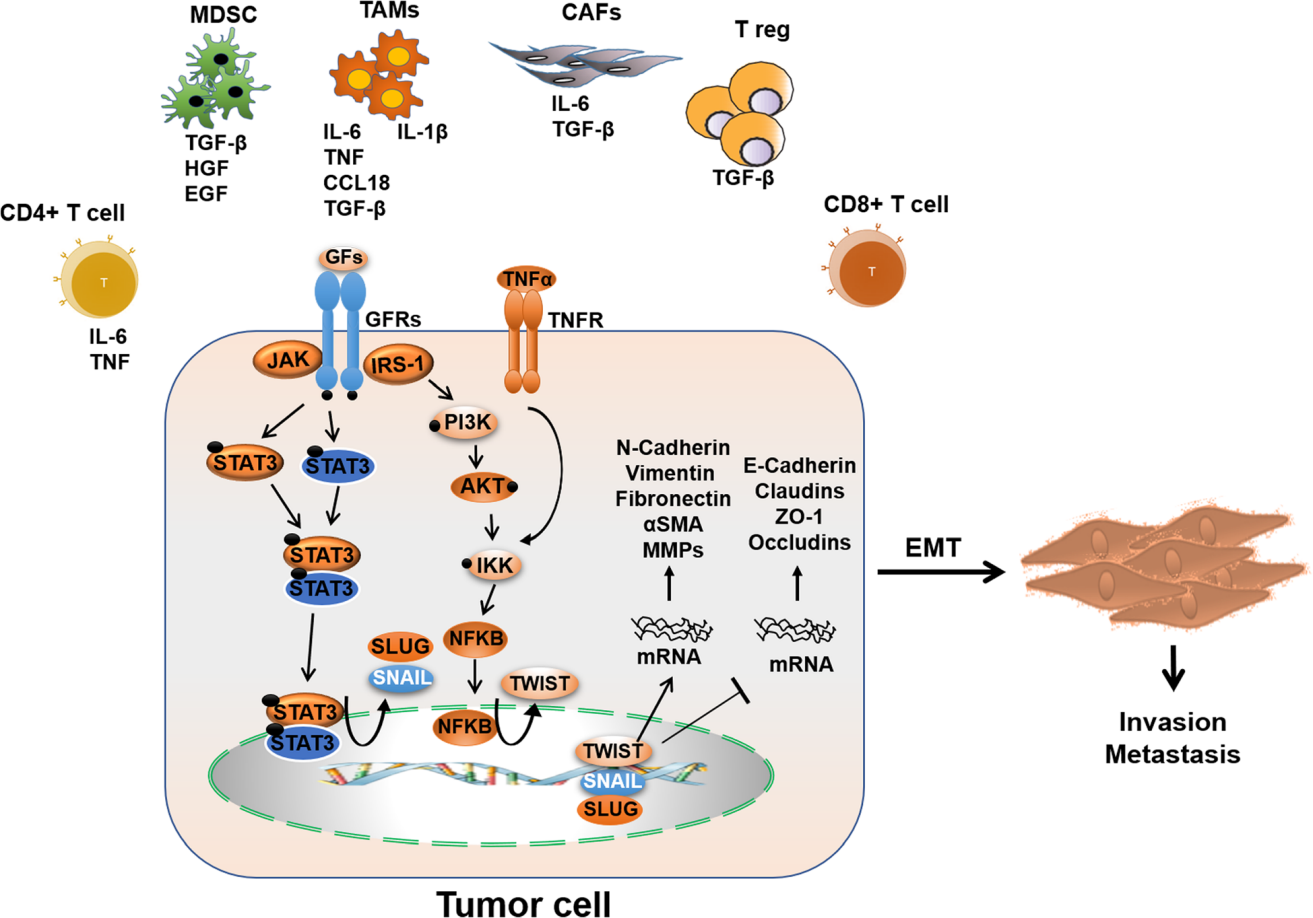
Complex network in TME orchestrating EMT. TME consists of TAMs, T cells, MDSCs, and CAFs. Through secretion of different cytokines and chemokines, it causes downregulation of epithelial marker, E-cadherin and upregulation of mesenchymal markers such as N-cadherin, vimentin, snail and ZEB1. In TME, these cells also upregulate cell signaling pathways such as STAT3, NF-kB and β-catenin, which all contribute to cell survival

EMT is an important cellular process in which epithelial cells acquire characteristics and morphology of mesenchymal cells [100, 101]. The transition from epithelial to mesenchymal phenomenon results in reduced expression of epithelial markers like E-cadherin and increased expression of mesenchymal markers such as ZEB2, vimentin, N-cadherin, and slug [102], contributing to cancer cell invasion and metastasis [103]. This phenomenon is a critical process in cancer development as the resulting mesenchymal cells gain aggressive phenotype by losing cell-cell adhesion and acquiring stemness, CRT resistance, and metastatic features [102, 104, 105]. Several cytokines and chemokines promote EMT in EC and are associated with poor prognosis and clinical outcome. N-cadherin is detected in non-epithelial cells and increases cell survival and migration, whereas E-cadherin suppresses the activation of cell survival pathways in epithelial cells [102]. As noted previously, IL-1β is highly expressed in EC tissues and promotes invasion and migration [75]. In vitro analysis has shown that IL-1β decreases E-cadherin expression, whereas it increases the expression of vimentin and snail in EC cells. These findings indicate that IL-1β could play an essential role in cancer metastasis. The release of IL-1β from M2 macrophages associated with increased expression (phosphorylation) of both NF-κB and IκBα suggests that IL-1β regulates EMT through the NF-κB pathway (Fig. 2) [75]. In addition to IL-1β, it has also been found that Monocyte Chemotactic Protein (MCP2 alias CCL8) secreted from M2 macrophages activates the NF-κB pathway and promotes migration and invasion of ESCC cells, thereby inducing the EMT process [106]. IL-6 and CXCR7 are also implicated in EMT through the NF-κB signaling pathway. High CXCR7 expression in response to IL-6 is associated with increased proliferation and chemoresistance in ESCC, a prominent feature of metastatic cells [107]. The inhibition of CXCR4 results in reduced expression of EMT genes in ESCC, which indicates that the increased expression of CXCR4 and CXCR7 are associated with poor prognosis in EC [108, 109]. Besides NF-κB signaling, IL-6/JAK2-STAT3 signaling also induces EMT by upregulation of EMT associated transcription factors [110–113].

Another interleukin, IL-6, is primarily involved in inducing EMT in ESCC and is associated with poor prognosis in patients [114, 115]. Serum levels of IL-6 were found to be considerably elevated in patients developing distant metastasis [115]. Also, the levels of IL-6 were significantly higher in the serum of ESCC patients who were chemoresistant compared to chemosensitive patients [107].

The acquirement of radioresistance by ESCC cells is one of the main factors leading to cancer recurrence and poor survival in ESCC. The IL-6/STAT3/TWIST signaling pathway inhibition is found to reverse ionizing radiation (IR)-induced EMT and radioresistance in ESCC [114]. Besides, the silencing of IL-6 was found to attenuate angiogenesis, reverse EMT, and increase cell death in ESCC [115]. Moreover, a study showed that treatment with CAF-derived IL-6 upregulated the expression of cancer stem cell markers and induced EMT transition, accompanied by increased migratory capacity in EAC cells [116]. In contrast, EAC tumors with reduced IL-6 expression exhibited epithelial phenotype [116]. Cells exposed to IL-6 show increased expression of mesenchymal markers such as vimentin, N-cadherin, CXCR4, ZEB1, and SNAI2 and reduced expression of epithelial markers such as E-cadherin, CD24, cytokeratin 19, CD29, ERBB2, and EPCAM, thus disseminating the role of IL-6 in the induction of mesenchymal characteristics in EAC [116].

Furthermore, the induction of IL-6 is found to increase the expression of ALDH1, a CSC marker, and TWIST, an essential EMT inducer, in a xenograft tumor model [117]. CSCs harbor increased EMT characteristics and are highly metastatic; therefore, IL-6 can promote EMT through increased ALDH1 expression in EC [118]. The elevated expression of MMP2, MMP7, MMP9, and vimentin and the reduced expression of E-cadherin in ALDH1A1^high^ ESCC indicates a close relationship between EMT and ALDH1A1 expression [119].

Another interleukin, IL-33, is a nuclear cytokine highly expressed in endothelial and epithelial cells [77]. Though IL-33 does not affect cell proliferation, it promotes invasion and migration of ESCC cells. Furthermore, it has been found that IL-33 promotes EMT and tumor progression by regulating the expression of CCL2 and recruiting Tregs through the TGF-β signaling pathway in ESCC [77]. In contrast, the knockdown of IL-33 is found to reduce the expression of EMT-related genes such as vimentin, N-cadherin, slug, and ZEB2, suggesting a role of IL-33 in EMT and metastasis [77]. Like IL-33, IL-23 is also involved in EMT and promotes invasion, migration, and metastasis of EC cells [120]. The underlying mechanisms revealed increased snail1 and slug expression [120].

While the molecular mechanisms underlying the cross-regulation between miRNAs and cytokines/chemokines in the promotion of tumor invasion and metastasis of ESCC remains largely unclear, but a study by Chen et al. showed that IL-23 promotes EMT, invasion, migration, and metastasis of EC cells by downregulation of miRNA 200a [120]. Several studies have reported that microRNA 200a plays a significant role in inhibiting EMT transformation, invasion, and metastasis by specifically targeting E-cadherin’s transcriptional repressors, ZEB1, and ZEB2 [121–124]. In patients with ESCC, members of the miR-200b cluster were shown to be significantly downregulated [125] and correlated dramatically with poor prognosis and adverse clinical pathology [125]. In vitro studies from the same group reported that miR-200b strongly represses cell invasiveness by modulating the cytoskeleton and the adhesive machinery in ESCC cells [126]. In contrast, ESCC patients with higher miR-200c expression responded poorly to chemotherapy and exhibited poor prognosis [126]. Besides, many cytokines are known to activate diverse signaling pathways that result in the deregulation of miRNAs, promoting tumor cell proliferation, invasion, metastasis, and escape from immune surveillance [127–131]. In addition to promoting EMT and immune surveillance, the interplay between cytokines/cytokines and miRNAs contributes to cancer-related inflammation (CRI) [132] mediated development and progression of ESCC [133]. In this direction, Zhang et al. reported the importance of miR-302b in inhibiting CRI in vitro and in vivo [134]. The mechanistic studies revealed decreased expression of transcription factors NF-kβ, STAT3, and HIF1α (important transcription factors in CRI) by miR-302b overexpression in TE11 cells and decreased tumor growth in vivo [134]. Also, STAT3 was downregulated by miR-124 in EC cells [135]. Furthermore, miR-302b also inhibited tumor growth by downregulating ErbB4 expression in ESCC [136]. These studies showed the complex intricacy of cytokine/chemokines and miRNA networks in regulating EC pathogenesis.

## Role of cytokine/ chemokine network in the development of metastatic niche

**Fig. 3:**
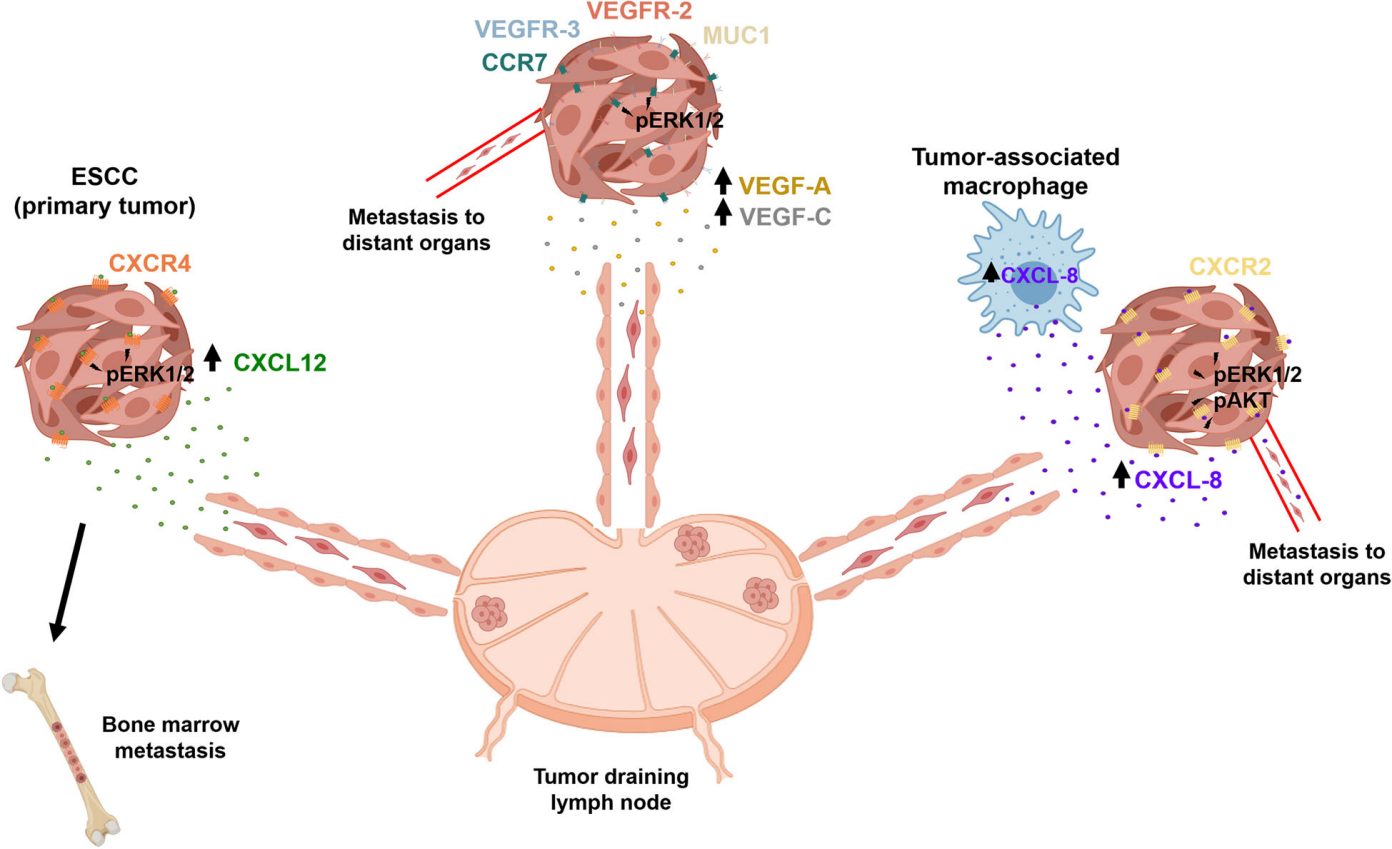
Role of cytokines/chemokines on tumor progression and dissemination. High expression of CXCL12/CXCR4 axis in primary tumor cells or/and ECSCs enhances the activation of p-ERK-1/2 and increases these cells’ ability to invade and metastasize to lymph node and bone marrow. Abundant expression of CCL21 in the endothelium of lymphatic vessels and lymph nodes attracts CCR-7-positive tumor cells. CCL21/CCR7 axis upregulates MUC-1 expression through p-ERK signaling and enhances the migration, invasion, and lymph node metastasis through the lymphatic system. High production of potent angiogenic (VEGF-A) and lymphangiogenic (VEGF-C) factors by primary tumor cells leads to a malignant process. Elevated levels of CXCL-8 secreted by primary tumor cells or TAMs induces the phosphorylation of AKT and ERK1/2 signaling pathway through the CXCR2 receptor expressed in primary tumor cells. These processes lead to further malignant progression to lymph nodes and distant organ metastasis through the lymphatic system and blood vessel. Also, circulating CXCL-8 contributes to the metastasis process

**Fig. 4:**
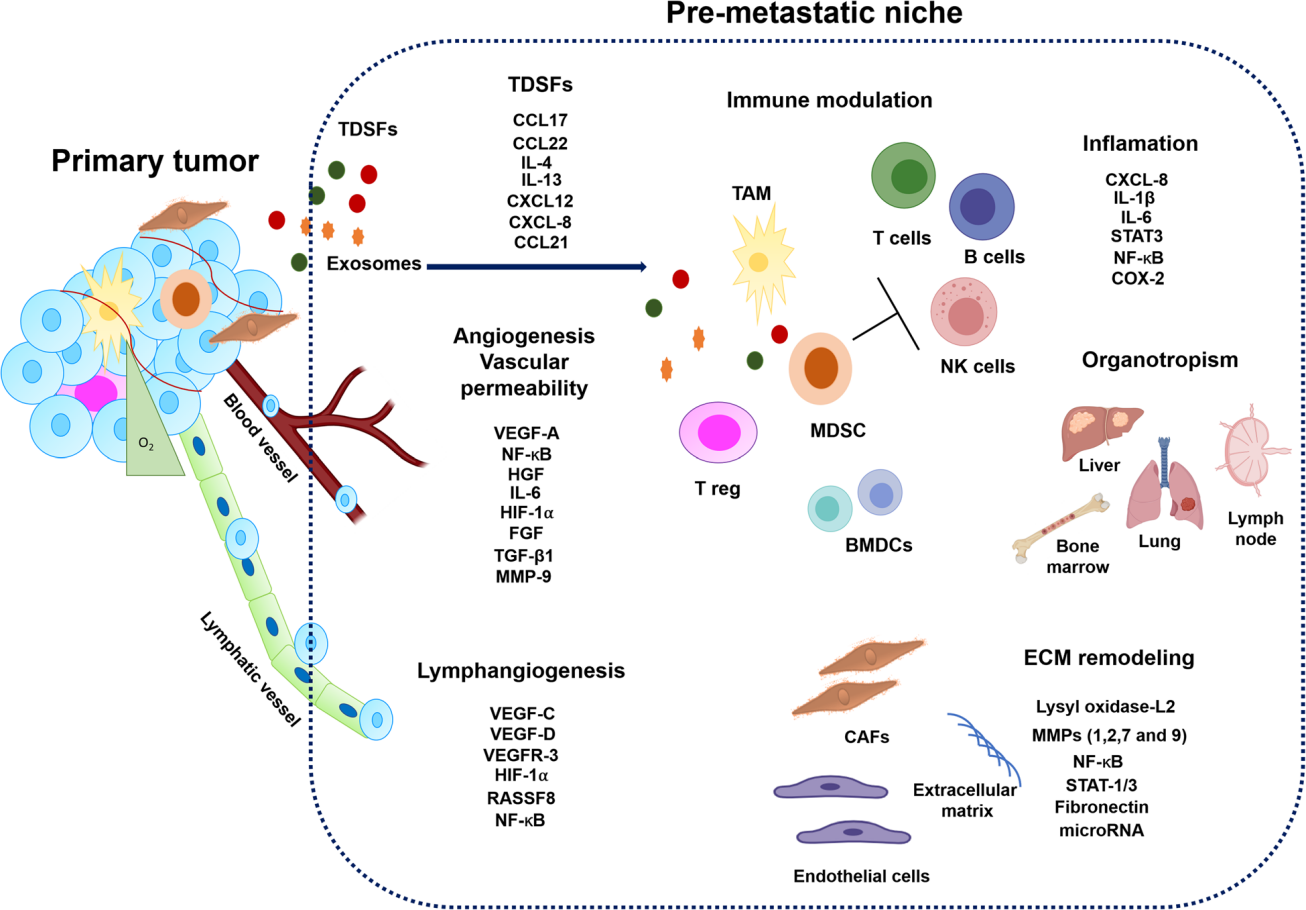
Role of Cytokines/chemokines in pre-metastatic niche formation Primary tumors establish a conducive microenvironment for eventual metastasis in secondary organs and tissue sites. A dynamic interplay between TDSFs, BMDCs, regulatory / suppressive immune cells, and stromal components in primary tumors provides an effective niche microenvironment for the tumor cells’ proliferation and metastasis. The specialized microenvironment dispels significant characteristic features that facilitate metastasis, such as inflammation, immunosuppression, ECM remodeling, angiogenesis, and lymphangiogenesis.

Metastasis is a multistep process requiring tumor cell detachment from the primary tumor and migration to target organs through the lymphatic or blood circulatory systems [137]. Specific organs are predisposed to metastasis in certain cancers (organotropism), and the formation of a supportive metastatic microenvironment determines tumor cell homing (Fig. 3). Accumulating lines of evidence suggest that primary tumors can prepare the microenvironment of distant organs for metastatic colonization [137]. Many studies have demonstrated that specific organs are predisposed to metastases in certain cancers due to the formation of a pre-metastatic niche in these organs (Fig. 4) [138]. Here, we will focus on the relevant cytokine/chemokine receptor axis to drive the metastatic process.

CXCL-12 is a chemokine that acts through CXCR4 and plays an essential role in tumor invasion and metastasis in numerous cancer types [139–141]. CXCR4 expression is elevated in tumor cells, and high levels of CXCL12 are expressed in tumor metastases target tissues such as lung, liver, brain, and bone [142]. Furthermore, CXCL12 activates tumor cells’ migration to target specific organs via a CXCL12-CXCR4 axis chemotactic gradient. Overexpression of CXCR4 was reported in both types of ESCC and EAD. Notably, the authors indicated that CXCR4 was expressed only in EC tissue but not in the normal esophageal epithelium, which suggests that expression of CXCR4 may be involved in the development of EC [143].

Additionally, positive expression of CXCR4 was associated with micrometastasis to both the lymph nodes and bone marrow with poor clinical outcome [143]. These results suggest that this receptor may have an essential role in the early metastasis spread in EC. These findings were in line with other studies, where CXCR4 expression in ESCC was significantly related to tumor grade, size, depth of tumor invasion, lymph node metastasis, and poor clinical outcome [101–103]. Koishi et al. reported that sustained upregulation of CXCR4 expression in ESCC clinical samples after CRT treatment contributed to tumor aggressiveness and a worse prognosis [144]. On the contrary, a previous study by Sasaki K et al. reported that patients with ESCC were positive for CXCR4 and/or CXCL12. Tumors that were positive for CXCL12 significantly correlated with advanced pathological features [145].

Several studies indicated that the CXCL12-CXCR4 signaling axis is essential for aggressive EC behavior and poor prognosis [141]. Gros et al. reported the homing efficiency of esophageal cancer cells that highly expressed CXCR4 to migrate to the liver, lung, peritoneum, and retroperitoneum after CXCL12 stimulation [146]. Recently, Wang X et al. reported a high expression of CXCR4 in esophageal cancer stem cells (ECSCs) from patients [147]. Additionally, in vitro studies have shown that ECSCs secreted high amounts of CXCL12 in an autocrine manner and increased its receptor expression CXCR4 compared with non-ECSCs [147]. Genetic and pharmacologic approaches that inhibit CXCR4 decreased the ability of ECSCs to invade and metastasize both in in vitro and in vivo models [147]. At the molecular level, ECSCs enhance the activation of the p-ERK1/2 pathway by the CXCL12/CXCR4 axis. The blockage of the ERK1/2 signaling pathway by CXCR4 or ERK1/2 inhibitors suppressed the ability of ECSCs to invade and metastasize [147]. These observations support a previous finding showing concomitant high expression of the CSC marker CD133 and CXCL4 in ESCC clinical samples [148].

CCR7 has been reported to support a metastatic niche [133] directly. CCR7 receptor activity is essential for immune cell entry into lymphatic vessels, and it binds to CCL21, preferentially expressed in secondary lymphoid tissues that drain many cancers [149]. Several studies indicate that high levels of CCR7 are associated with tumor metastasis and a poor clinical outcome in several cancer types, including EC [150, 151]. Accordingly, an in vivo study showed that tumor cells with high expression of CCR7 had higher metastatic potential in the lymph node of the heterotopic transplantation mouse model [99]. These findings confirmed that CCR7 expression is an essential predictor of lymph node metastasis and poor prognosis of ESCC patients’ survival. A critical study showed the co-expression of CCR7 and MUC1 in ESCC clinical samples [97]. In this study, patients who co-expressed CCR7 and MUC1 had increased lymph node metastasis, regional lymphatic recurrence, and lower survival rates [97]. These results revealed that CCR7 and MUC1 display a significant cross-talk in metastasis progression in this type of tumor. Mechanistically, the CCL21-CCR7 axis upregulated MUC1 expression through the activation of the ERK1/2 signaling pathway in ESCC cells in vitro [97]. In addition, the knockdown of MUC1 significantly suppressed the migration and invasion of ESCC cell lines induced by treatment of the cells with CCL21 [97]. These results confirmed that the CCL21/CCR7 axis enhances the migration and invasion of cancer cells via the ERK1/2 signaling pathway and that these findings are consistent with earlier observations [152]. Song et al. demonstrated that co-expression of CCR7 and VEGF-C correlated with higher lymphatic metastatic recurrence in patients with pN0 ESCC [153]. The authors suggested that cross-talk occurs between CCR7 and VEGF-C, and their expression may be used to predict metastatic lymphatic recurrence of pN0 ESCC [153]. Indeed, several studies have described the close correlation between high VEGF-C expression in tissue and serum samples with tumor differentiation, depth of the tumor, vascular and lymph node metastasis, lymphatic invasion, tumor stage, and a decreased survival rate in ESCC clinical samples [154, 155]. VEGF-C can mediate the upregulation of CCL21 secretion in lymphatic endothelium, which drives CCR7-dependent tumor chemo invasion towards lymphatics [156]. In addition to VEGF-C, studies have demonstrated the correlation between VEGF-A expression in both tissue and serum samples with clinicopathological factors in ESCC patients [157, 158]. VEGF-A and VEGF-C induce a premetastatic niche in regional lymph nodes by activating lymphangiogenesis to enhance distant metastasis [159, 160]. In addition, activation of the VEGF-C and PI3K signaling is essential for remodeling the lymph nodes before tumor cells’ arrival by stimulating lymphatic endothelial cell proliferation, leading to tumor-associated lymphangiogenesis [161]. These results confirmed that the angiogenic factors, VEGF-A and VEGF-C, are involved in both primary tumor growth and the development of a metastatic niche in ESCC.

CXCL8 is another interleukin that plays an essential role in tumor metastasis. CXCL8 was associated with tumor progression in different tumor types [89, 162]. Multiple studies have shown that CXCL8 overexpression in ESCC patients is related to lymph node metastasis and worse prognosis [57, 163]. Krzystek-Korpacka showed that circulating CXCL8 was significantly elevated in ESCC patients and associated with tumor size, cancer dissemination, lymph node presence, and distant metastasis [164]. They also found that circulating CXCL8 positively correlated with platelets and leukocyte levels and biochemical markers of inflammation, such as C-reactive protein [164].

Similarly, Hannelien et al. reported that infiltrated neutrophils could also produce CXCL-8 and induce proliferation of epithelial cells, facilitating the progression of Barrett’s esophagus and EC [165]. These results might suggest a relationship between CXCL8 chemokine and inflammation in ESCC. However, the authors concluded that detection rates were not satisfactory enough to allow for the recommendation of CXCL8 testing as an adjunct to the clinical evaluation of lymph node involvement in ESCC patients [164]. In another study, Ogura et al. reported that overexpression of CXCL8 and its receptor CXCR2 in ESCC clinical simples significantly correlated with adverse pathological features [57]. Indeed, CXCR2 is highly expressed in ESCC patients and is significantly associated with lymph node metastasis and a reduced overall survival rate [163].

Immunohistochemistry and immunofluorescence study of tissue samples in patients with ESCC revealed elevated expression of CXCL8 and increased infiltration of TAMs significantly correlated with lymph node metastasis and bad prognosis [166]. The authors [166] also observed that CXCL8 was upregulated in peripheral blood monocytes (PBMo)-derived macrophages stimulated with conditioned media of TE-series ESCC cell lines (TAM-like PBMo-derived macrophages), which facilitated the migration and invasion of ESCC cells by inducing the phosphorylation of the AKT and ERK1/2 signaling pathway through activation of CXCR1 / CXCR2 receptors. In this context, neutralizing antibodies against CXCR1, CXCR2, or CXCL8 suppressed the migration and invasion in a human ESCC cancer cell model induced by CXCL8 [166]. Moreover, the neutralizing antibody against CXCR2 demonstrated a higher suppressive effect on the migration and invasion in ESCC cell lines activated by TAM-like PBMo-derived macrophages compared to the neutralizing antibody against CXCR1 and CXCL8 [166]. These observations confirm a previous finding that CXCL8 binds to CXCR1 / CXCR2 and activates the signaling pathways for AKT and ERK1/2 in other cancer cell lines [167]. Clinical data indicated that CXCL8 expression was significantly correlated with disease-free survival (DFS), lymph node metastasis, high expression levels of CXCR2, and high infiltration of an increased number of CD204-positive macrophages [166]. CD204-positive macrophage infiltration was strongly associated with pathological factors in ESCC patients [42]. Overall, these findings indicated that CXCL8 could be involved in the growth of ESCC as an autocrine/paracrine factor by increasing the migration and invasion of tumor cells. Also, blocking the CXCL8 and its receptors (CXCR1 / CXCR2) appears to be a novel therapeutic strategy that may reverse the tumor cell metastatic phenotype in patients with ESCCs.

## Role of cytokine/ chemokine network in escape from immune surveillance

**Fig. 5:**
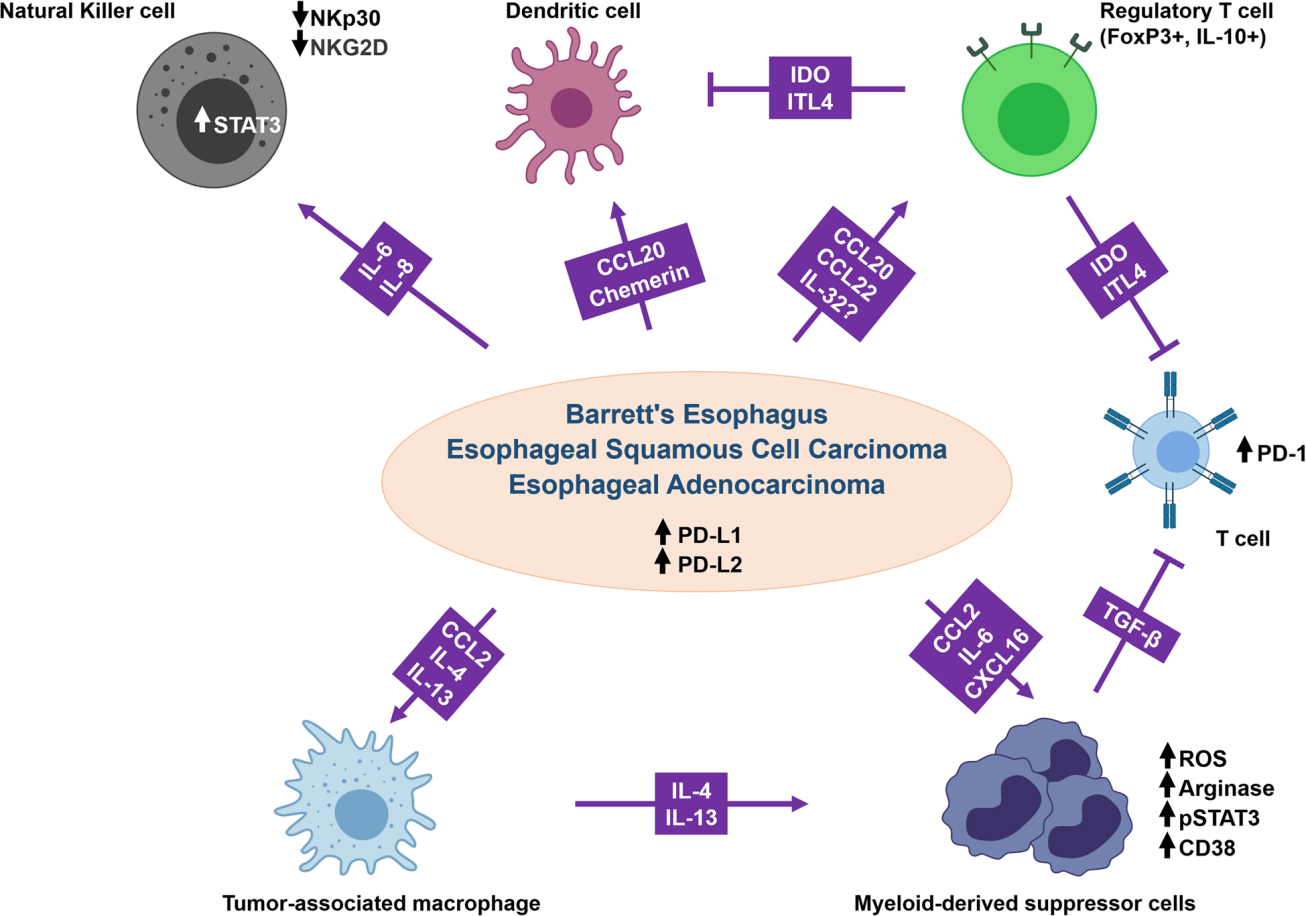
Tumor evasion from immune surveillance mediated by cytokines/chemokines in the TME. Cancer cells secrete several chemokines and cytokines that inhibit NK cells, DCs, and T cell functions and recruit TAMs. Tumor cells also induce MDSCs and Treg T cells that can further inhibit T cell function. TAMs and tumor cells both express PD-L1/2 to inhibit T-cell activation via the PD-1 receptor. Altogether, these cells suppress antitumor immunity while also promoting tumor growth and progression by various mechnisms

Inflammation is a crucial feature of the TME, underlying the concept that tumors are ‘wounds that do not heal’ [168]. Tumors undergo inflammation to escape immune surveillance and sustain tumor growth by converting immune cells into immunosuppressive entities within the TME (Fig. 5). The infiltration of immune cells into the tumor is controlled by a complex network of pro-inflammatory cytokines involved in immune dysfunctions, thus contributing to tumor growth and progression [169]. Indeed, high levels of expression of several immunosuppressive cytokines have been reported in EC, as mentioned in previous sections. Likewise, increased serum IL-8 levels are related to lymph node and distant metastases in ESCC [164]. Immune gene expression profiling of EAC tumors revealed that the chemokine-receptor axes CXCL9, − 10,-11/CXCR3 are prominent in EAC, most likely promoting cancer cell proliferation and metastasis [170].

Furthermore, lower levels of CCL11 and CXCL10 in post-therapeutic esophageal tumor tissues were associated with a better prognosis [21]. Ultimately, the accumulation of pro-inflammatory cytokines triggers an immunosuppressive TME leading to the attenuation of the innate and adaptive immune responses. NK cells are innate immune cells that display strong cytolytic function against tumor cells. However, their function can be hampered by the TME [171]. Indeed, the expression of IL-6 or IL-8 in ESCC tumor tissues was found to impair NK cell function and positively correlated with tumor progression and poor survival. Mechanistically, primary ESCC cells activated the STAT3 signaling pathway on NK cells through IL-6 and IL-8 secretion, leading to the downregulation of activating receptors (NKp30 and NKG2D) on the surface of NK cells [172]. Likewise, disturbing dendritic cell (DC) functions, which are specialized antigen-presenting cells playing critical roles in the priming of effector T-cells, is a well-described mechanism of antitumor immunity inhibition [173]. Although DCs play a key role in inducing and maintaining the antitumor immunity, a broad spectrum of cells and factors in the TME are known to deliver differential stimuli that affects the functional plasticity of DCs [174] and condition them to function towards immune tolerance or immunosuppression [175]. EC cells can promote dendritic cells’ recruitment during the esophageal metaplasia-dysplasia-carcinoma sequence through the secretion of two chemoattractants: CCL20 (also known as macrophage inflammatory protein 3α) and chemerin. A co-culture of dendritic cells with BE and EAC was found to convert the dendritic cells to a tolerogenic phenotype and to stimulate Treg cells differentiation from naïve T cells [176]. Within the TME, Treg cells are involved in the inhibition of the antitumor immunity by triggering immune suppressive mechanisms leading to tumor development and progression [177]. Recent studies suggest that Treg cells are preferentially trafficked to the TME in response to chemokine gradients production by the tumor. A high infiltration by Treg cells is associated with poor survival in several types of cancers [178]. Indeed, several chemokines correlated with Treg cells infiltration in ESCC. For instance, Liu et al. showed that Treg cells migrate towards CCL20 in vitro in a Transwell chemotaxis assay and that CCL20 secretion positively correlated with Treg cells markers expression (FoxP3 and IL-10) in ESCC tumors [179].

Interestingly, the authors also found that intratumoral expression of CCL20 predicts ESCC patients’ survival [179]. Similarly, CCL22 expression in squamous cell carcinomas of the upper aerodigestive tract, including ESCC, correlated with Treg density in the tumors (FoxP3+). Treg cells’ density also correlated to the serum level of the Treg cells-associated inhibitory cytokine IL-10, indicating the presence of IL-10-associated cellular immunosuppression in these patients [180, 181]. Moreover, the multivariate analysis suggested that a combination of IL-32 expression in ESCC and Treg cell infiltration was associated with poor survival [43]. IL-32 might contribute to the antitumor immune response damping by inducing the secretion of immunosuppressive molecules such as IDO and ILT4, previously described in CD4+ T cells, CD163+ macrophages, Treg cells, and DC [182].

The accumulation of TAM represents another major cellular component of the immunosuppressive TME. TAM actively participates in establishing and maintaining an immunosuppressive TME by producing cytokines, chemokines, growth factors, and triggering the inhibitory immune checkpoints in T cells [183]. Some studies have shown that the CCL2-CCR2 axis predicted poor prognosis in ESCC patients and correlated with TAM accumulation in esophageal carcinogenesis [41, 184]. More importantly, using a mouse model of ESCC, M2 polarization of TAM increased PD-L2 expression in these cells, resulting in immune evasion and tumor promotion through a PD-1 signaling pathway. This was most likely a result of the PD-L2 presentation [184]. Moreover, the high density of TAMs in esophageal cancer tissues was associated with increased PD-L1 expression and significantly worse overall survival (ref). In vitro assays demonstrated that the co-culture of EC cell lines with activated M2-like TAM increased cell invasion and migration abilities of the cancer cells as well as elevated PD-L1 expression in these cells [185].

Similarly, PD-L2 expression was abundant in EAC as well as BE cells [186]. Furthermore, the Th2 cytokines, IL-4, and IL-13, associated with the Th2 immune response accompanying BE, induced PD-L2 expression in EAC cell lines. These results suggested that the inflammatory environment in BE and EAC may contribute to the expression of PD-L2 and promote immune evasion in EAC through the PD-1 signaling pathway [186]. The same Th2 cytokines were also found to be not only associated with M2 macrophages polarization and infiltration of CD163^+^ M2 TAM in EAC tissues, but also with the presence of circulating MDSC and plasma arginase 1, which is supposed to promote the formation of the immunosuppressive microenvironment in EAC patients [187].

The accumulation of immature MDSC with potent immunosuppressive activity is typical in tumors, where they can differentiate into mature myeloid cells such as TAMs and DCs [188]. In EC, Chen et al. have reported that circulating MDSCs were significantly increased in ESCC patients and are associated with increased IL-6 levels in the blood. The association of MDSC and IL-6 is linked with poor prognosis in patients with ESCC [189]. Using an esophageal tumor animal model, the authors confirmed that MDSC recruitment was associated with invasive esophageal tumors and increased IL-6 levels. IL-6 was shown to promote the immunosuppressive functions of MDSC by stimulating the production of ROS and arginase and p-STAT3 expression in MDSCs [189]. Likewise, using a murine model of oral-esophageal cancer, CD38 was identified as playing a central role in MDSC immunosuppressive functions. Indeed, MDSC with higher CD38 expression displayed a greater capacity to suppress activated T cells and to promote tumor growth than MDSC with lower CD38 expression. Of note, IL6, IGFBP3, and CXCL16 were identified as the tumor-derived factors responsible for the induction of CD38 expression in MSDC ex vivo [190]. More recently, MDSC from ESCC patients were shown to secrete TGF-β, which in turn increased PD-1 expression on tumor-infiltrating CD8+ T cells and enhanced their resistance to PD-1/PD-L1 blockade. Interestingly, blocking the TGF-β signaling pathway restored the proliferation of antigen-specific CD8+ T cells suppressed by MDSC. Furthermore, dual blockade of PD-1/PD-L1 and TGF-β enhanced the antitumor ability of antigen-specific CD8+ T cells in vivo [191].

## Targeting cytokine/ chemokine network in esophageal cancer

The changes in the expression of chemokines and cytokines in pathological conditions influence recruitment and activation of immune cells, tumor cell proliferation, and metastasis. Since chronic inflammation plays a crucial role in EC progression and metastasis, there has always been a great interest in inhibiting the chemokine- cytokine signaling in cancer. However, the biological functions carried out by these molecules make it tricky to find a suitable methodology to selectively inhibit chemokine- cytokine pathway in tumors while creating minimal disturbances to the immune cell responses with manageable side effects.

CXCL12 and its receptor, CXCR4, are among the most studied chemokine/chemokine receptors in the scenario of cancer metastasis. The CXCL12-CXCR4 axis regulates critical aspects such as cancer cell proliferation, chemotaxis, and invasion. Patients with CXCL12 positive tumors had drastically reduced overall survival and disease-free survival rates [147]. Wang et al. [147] have shown that the ECSCs from clinical samples have overexpression of CXCR4 along with the capability for CXCL12 autocrine secretion. The blockage of CXCR4 using small molecule inhibitor (AMD-070) or RNA interference (using specific shRNA) significantly reduced the ability of EC stem cells to invade and metastasize in in vitro (trans-well migration and matrigel invasion) and in vivo models (mouse caudal vein tumor xenograft model). The CXCL12-CXCR4 mediated activation of extracellular signal-regulating kinase 1/2 promotes invasion and metastasis in EC stem cells [147]. Simultaneously, silencing the expression of CXCR4 using lentiviral shRNA has been shown to inhibit EC cell growth and induce apoptosis in vitro and in vivo [192].

Since the CXCL12-CXCR4 axis is essential for cancer metastasis, many CXCR4 antagonists are in clinical use/development for cancer therapy. Of these, plerixafor has already been approved for mobilizing hematopoietic stem cells, while a phase II clinical trial is in progress to evaluate its use in combination with standard temozolomide chemo-radiotherapy for patients with glioblastoma (NCT03746080). Plerixafor and Granulocyte-colony stimulating factor combination has also been shown to mobilize hematopoietic stem cells more efficiently than plerixafor alone [193]. Plerixafor is a bicyclam molecule that selectively and reversibly antagonizes the binding of stromal cell-derived factor-1 on bone marrow stromal cells’ surface to chemokine CXCR4 on hematopoietic stem cells, with their subsequent mobilization in the blood [193]. As of now, no clinical trials are going on in esophageal cancer with plerixafor and could be the focus of future research. The detailed clinical trial targets in EC are described in Table 1.

**Table 1:** Clinical trial targets in esophageal cancer

Clinical trial identifier	Trial Drug	Target	Patient population	Phase	Findings	References
NCT02054806	Pembrolizumab	PD-1	PD-L1 positive ESCC; EAC; GEAC; GESCC	I	Delayed tumor progression and durable anti-tumor activity with manageable toxicity	[194]
NCT02559687	Pembrolizumab	PD-1	Metastatic/advanced ESCC; EAC; Siewert type 1 GEAC	II	Durable anti-tumor activity with a manageable safety profile	[195]
NCT02564263	Pembrolizumab	PD-1	Metastatic/advanced ESCC; EAC; Siewert type 1 GEAC	III	Improved overall survival in combination with chemotherapy drugs (irinotecan, paclitaxel, docetaxel), minimum toxicity	[196]
NCT02971956	Pembrolizumab	PD-1	Metastatic EAC; Siewert type 1 GEAC	II	Active drug in previously treated esophageal cancer patients with a favorable safety profile	[197]
ONO-4538-07/JapicCTI-No.142422	Nivolumab	PD-1	Treatment-refractory ESCC; EAC; AC in the cervical or thoracic esophagus	II	Reduced tumor burden with a manageable safety profile	[198]
NCT01928394	Nivolumab + ipilimumab	PD-1/CTLA4	Chemotherapy-refractory metastatic EAC; GAC; GEAC	I/II	Durable antitumor activity and improved overall survival with a manageable safety profile	[199]
NCT02954536	pembrolizumab + trastuzumab + capecitabine + oxaliplatin	PD-L1	HER2+ metastatic esophagogastric AC	II	Tumor regression, improved objective response rate with few immune related toxicities	[200]
NCT02120911	Trastuzumab + pertuzumab	HER2	HER2+ EAC	I/II	Improved overall survival and treatment response	[201]
ISRCTN29580179	Gefitinib	EGFR	ESCC; EAC	III	No improvement in overall survival but possess palliative benefits for patients with a short life expectancy	[202]
NCT01336049	Nimotuzumab + cisplatin + paclitaxel	EGFR	Metastatic ESCC	II	Improved progression-free and overall survival in patients with the unresectable local regional disease and metastatic disease	[203]
UMIN000003557	HLA-A-24-restricted epitope peptides URLC10, CDCA1, KOC1 mixed with montanide	CD8+ T cell	Thoracic ESCC	II	Improved relapse-free survival in patients who showed CD8^+^ T cell induction to multiple peptides	[204]
NCT00682227	HLA-A24-restricted epitope peptides TTK, LY6K, IMP-3 mixed with montanide	T-cells	ESCC	I	Strong induction of antigen-specific T-cell responses with satisfactory safety and good immunogenicity	[205]
UMIN000010158	adenovirus 5 vector OBP-301 (Telomelysin)	T-cells	EC	I/II	complete response in 2 patients and partial response with tumor regression in 1 patient with manageable tolerance	[206]
NCT00917384	Ramucirumab	VEGFR-2	Advanced GAC; GEAC	III	Prolonged overall survival and reduced disease progression risk	[207]
NCT01170663	Ramucirumab + paclitaxel	VEGFR-2	Advanced GAC; GEAC	III	Increased overall survival	[208]
NCT01472016	ABT-700	c-MET	Advanced GEC	I	MET amplification was more common in treatment-refractory tumors	
NCT01611857	Tivantinib + FOLFOX	c-MET	Metastatic EAC;GEAC; AC of the stomach	I/II	The treatment showed response and progression-free survival with few treatment-related toxicities	[209]
NCT00909025	IMAB362 (Zolbetuximab)	Claudin 18.2	Advanced GAC; GEAC	I	Well tolerated drug with a favorable safety profile	[210]
NCT01630083	IMAB362 in combination with EOX (Epirubicin, Oxaliplatin, Capecitabine) chemotherapy	Claudin 18.2	Advanced GAC; GEAC; EAC	II	Prolonged overall survival	[211]
NCT02013154	DKN-01 + Paclitaxel	DKK1	Refractory or relapsed EAC; GEAC	I	Combination of DKN-01 + Paclitaxel showed tolerance with favorable safety profile	[212]
NCT01795768	AZD4547	FGFR	Advanced GEAC	II	Inhibited FGFR2 amplification	[213] (Abstract)
NCT00632333	TTK + URLC10 + KOC1 + VEGFR1 + VEGFR2 with concurrent cisplatin and fluorouracil	HLA-A*2402	ESCC	I	The combination therapy proved to be well-tolerated with few toxicities and a satisfactory safety profile	[214]
NCT01612546	CRLX101	DNA Topoisomerase I	Chemotherapy-refracted EAC	II	Exhibited minimal activity with stable disease and favorable toxicity profile	[215]

Abbreviations: *ESCC* Esophageal Squamous Cell Carcinoma, *EAC* Esophageal Adenocarcinoma, *GAC* Gastric Adenocarcinoma, *GEC* Gastroesophageal Cancer, *GEAC* Gastroesophageal Junction Adenocarcinoma, *AC* Adenocarcinoma; GESCC, gastroesophageal squamous cell carcinoma

BL-8040/ Motixafortide is a short high-affinity synthetic peptide antagonist of CXCR4 with long receptor occupancy undergoing a phase Ib / II trial (NCT02826486). This trial investigates the safety, pharmacokinetics, and anti-cancer activity as a combination immunotherapy in patients with locally advanced or metastatic gastric /gastroesophageal junction cancer/ esophageal cancer.

Dysplastic and malignant lesions demonstrate promoter demethylation and gene amplification in chromosome 4q21 cluster containing genes for the chemokines CXCL8, CXCL1, and CXCL3; all are overexpressed in BE [216]. CXCL8 and its receptor CXCR2 (IL-8 Receptor, beta) are overexpressed in ESCC, and their level correlates with lymphatic invasion, venous invasion, lymph node metastasis, depth of invasion, and poor prognosis [57]. Simultaneously, ESCC patients have higher levels of circulating CXCL8 compared to healthy controls, and the level correlates with tumor size and metastasis [164]. Shrivastava et al. have reported that blockade of CXCR2 using a highly specific small-molecule inhibitor, SB332235 can significantly reduce matrigel invasion capacity of the human EC cell line OE33 without having any effect on cell proliferation. Similarly, Wu et al. showed that silencing CXCR2, using small interfering RNAs, significantly reduced cell invasion while silencing CXCR7 did not affect cell invasion. Silencing both chemokines resulted in reducing cancer cell viability and increased induction of apoptosis [88]. These studies show the potential of CXCR2 as a therapeutic target for managing metastasis in EC.

IL-1 family members, IL-1α and IL-1β, are known to promote cancer cell proliferation, invasiveness, and metastasis. These can induce the expression of several growth factors and angiogenic genes [70]. Due to these effects, several blockers of IL-1 signaling were developed for the management of advanced solid tumors and hematological malignancies. IL-1 receptor antagonist (IL-1RA) is a member of the IL-1 family that blocks IL-1α / IL-1β signaling by binding to the same receptor [70]. In vitro studies have shown that stimulation of human EC cell lines with IL-1β increases invasiveness while blocking it using anti-IL-1β antibody reduces cell invasion (reviewed in [70]). Treatment of EC cell lines with caffeic acid phenethyl ester, a specific inhibitor of NF–κB, inhibited cell migration and invasion in vitro, and reduced tumor growth in vivo [70]. Several studies have shown that IL-1RA expression is significantly lower in ESCC patients’ samples [74, 217] than adjacent normal tissues. The reduced expression of IL-1RA correlated with advanced clinical staging of the tumor, decreased 5-year survival, and poor clinical outcome. Chen et al. has shown that overexpression of IL-1RA can reduce the proliferation of EC cell line with constitutive expression of IL-1 α, albeit without any effect on cell migration as observed by scratch wound assay [70].

Furthermore, the expression of IL-6 is found to correlate with distant metastasis positively and negatively correlate to treatment response. Chen et al. showed that inhibition of IL-6 using shRNA resulted in a significant reduction in human EC migration and invasion in vitro and reduced tumor xenograft growth in a mouse model [115]. Similarly, Ebbing et al. have reported that the CAFs from EC patients secrete biologically active IL-6, which drives resistance to chemo- and radiotherapy, induces EMT, and enhances the migratory and clonogenic capacity of EC cells. Also, the inhibition of IL-6 using a neutralizing antibody was found to significantly reduce the migratory and clonogenic capacity of EC cells [116]. No clinical studies were carried out to evaluate the ability to target IL-6 in ECs even though anti-IL-6 antibodies such as siltuximab, tocilizumab, sarilumab, etc. have been approved by the food and drug administration (FDA) for managing several other malignancies.

## Conclusion and future perspectives

The findings in this review suggest that the cytokine/chemokine network contributes to the aggressiveness of ESCC and correlates with primary tumor progression, lymph and distant metastasis, and patient outcome. This review provides novel insight into the mechanisms of dynamic interaction and cross-talk between the tumor cells and components of the TME in EC. The research into the cancer cytokine/chemokine network has revealed a diverse range of changes occurring in the local immune response, tissue microenvironment, metabolic profile, intracellular signaling mechanisms that contribute to tumor growth and progression. The findings presented in this review reveal the potential of disrupting the interaction between tumor cells and TME components for ESCC therapy. Although the therapeutic approaches mentioned herein showed a reduction in tumor growth rates, they remained short of eliminating the tumor. Therefore, future breakthroughs in cancer therapy will likely depend on improvements in drug design and combination therapies that target inflammatory cytokine/chemokine networks. It has been shown that IL-6 mediates cross-talk between tumor cells and the TME by supporting tumor cell growth and promoting fibroblast activation. As a result, IL-6 receptor (IL-6Rα) and downstream effectors offer targeted therapy opportunities in ESCC. To this end, a new generation of IL-6Rα targeting antibodies is rationally developed in such a way to specifically block IL-6 trans-signaling, considered to be more relevant to carcinogenesis.

In contrast to targeting the receptors, it could be beneficial to target downstream components of the signaling pathways that these receptors activate. For example, combining small molecule inhibitors of STAT3 and MEK/ERK could be a successful strategy. This approach can decrease cancer cells’ ability to acquire resistance to targeted therapies and potential compensation by other cytokines in the TME. Although inflammatory cytokine /chemokine networks play an essential role in tumor proliferation, angiogenesis, and metastasis, their mechanisms of action in EC are not entirely explained. Further studies are necessary to establish the biological significance of cytokine/chemokine networks in EC and their potential usefulness as future drug targets.

## Data Availability

Not Applicable.

## Abbreviations

EC: Esophageal cancer
ESCC: Esophageal squamous cell carcinoma
TME: Tumor microenvironment
EMT: Epithelial to mesenchymal transition
BE: Barrett’s esophagus
GERD: Gastroesophageal reflux disease
AP: Apurinic/apyrimidinic
BER: Basic excision repair
ECM: Extracellular matrix
NK: Natural killer
Treg: Regulatory T cells
DC: Dendritic cells
CRT: Chemoradiation therapy
CAFs: Cancer-associated fibroblasts
MDSCs: myeloid-derived suppressor cells
TAMs: Tumor-associated macrophages
PD-1: Programmed cell death protein-1
MIF: Migratory inhibition factor
TNF-α: Tumor necrosis factor α
ROS: Reactive oxygen species
EAC: Esophageal adenocarcinoma
CSCs: Cancer stem cells
ECSCs: Esophageal cancer stem cells
DFS: Disease-free survival
MCP-1: Monocyte chemoattractant protein-1
VEGF: Vascular endothelial growth factor
APE1: Apurinic/apyrimidinic endonuclease-1
REF-1: Redox effector factor-1
BMDCs: Bone marrow-derived cells
TDSFs: Tumor-derived secreted factors

## References

[1] Napier KJ, Scheerer M, Misra S (2014). Esophageal cancer: a review of epidemiology, pathogenesis, staging workup and treatment modalities. World J Gastrointestinal Oncol.

[2] Rustgi AK, El-Serag HB (2014). Esophageal carcinoma. N Engl J Med.

[3] Valverde CM (2006). Novel targets in gastric and esophageal cancer. Crit Rev Oncol Hematol.

[4] Kuwano H (2003). Distinctive clinicopathological characteristics in esophageal squamous cell carcinoma. Ann Thoracic Cardiovasc Surg.

[5] Kumagai Y (2010). Angiogenesis in superficial esophageal squamous cell carcinoma: magnifying endoscopic observation and molecular analysis. Dig Endosc.

[6] Mariette C, Piessen G, Triboulet J-P (2007). Therapeutic strategies in oesophageal carcinoma: role of surgery and other modalities. Lancet Oncol.

[7] Bystricky B, Okines AFC, Cunningham D (2011). Optimal therapeutic strategies for Resectable Oesophageal or Oesophagogastric junction Cancer. Drugs.

[8] Messmann H (2001). Squamous cell cancer of the oesophagus. Best Pract Res Clin Gastroenterol.

[9] Reid BJ (2010). Barrett's oesophagus and oesophageal adenocarcinoma: time for a new synthesis. Nat Rev Cancer.

[10] Pera M (2005). Epidemiology of esophageal adenocarcinoma. J Surg Oncol.

[11] Bernstein H (2009). Bile acids as endogenous etiologic agents in gastrointestinal cancer. World J Gastroenterol.

[12] Jenkins GJ (2008). The bile acid deoxycholic acid has a non-linear dose response for DNA damage and possibly NF-kappaB activation in oesophageal cells, with a mechanism of action involving ROS. Mutagenesis.

[13] Inayama M (2007). Involvement of oxidative stress in experimentally induced reflux esophagitis and esophageal cancer. Hepatogastroenterology.

[14] Zhang HY (2009). In benign Barrett's epithelial cells, acid exposure generates reactive oxygen species that cause DNA double-strand breaks. Cancer Res.

[15] Dvorak K (2007). Bile acids in combination with low pH induce oxidative stress and oxidative DNA damage: relevance to the pathogenesis of Barrett's oesophagus. Gut.

[16] Hong J (2016). APE1-mediated DNA damage repair provides survival advantage for esophageal adenocarcinoma cells in response to acidic bile salts. Oncotarget.

[17] Fishel ML, Kelley MR (2007). The DNA base excision repair protein Ape1/Ref-1 as a therapeutic and chemopreventive target. Mol Asp Med.

[18] Tell G (2009). The many functions of APE1/Ref-1: not only a DNA repair enzyme. Antioxid Redox Signal.

[19] Bhat AA (2018). Exposure of Barrett's and esophageal adenocarcinoma cells to bile acids activates EGFR-STAT3 signaling axis via induction of APE1. Oncogene.

[20] Lu H (2019). APE1 Upregulates MMP-14 via redox-sensitive ARF6-mediated recycling to promote cell invasion of esophageal adenocarcinoma. Cancer Res.

[21] Blank S (2017). Inflammatory cytokines are associated with response and prognosis in patients with esophageal cancer. Oncotarget.

[22] Diakowska D (2013). Cytokines association with clinical and pathological changes in esophageal squamous cell carcinoma. Dis Markers.

[23] Li Z (2019). Clinical significance of serum chemokines in esophageal Cancer. Med Sci Monit.

[24] Ponomarev AV, Shubina IZ (2019). Insights into mechanisms of tumor and immune system interaction: association with wound healing. Front Oncol.

[25] Lin EW (2016). The tumor microenvironment in esophageal cancer. Oncogene.

[26] Balkwill F, Mantovani A (2001). Inflammation and cancer: back to Virchow?. Lancet.

[27] Blank S (2015). Angiogenic and growth factors in gastric cancer. J Surg Res.

[28] Wilson J, Balkwill F (2002). The role of cytokines in the epithelial cancer microenvironment. Semin Cancer Biol.

[29] Durand RE (1991). Keynote address: The influence of microenvironmental factors on the activity of radiation and drugs. Int J Radiat Oncol Biol Phys.

[30] Lee H-J (2014). Drug resistance via feedback activation of Stat3 in oncogene-addicted Cancer cells. Cancer Cell.

[31] Lee K-W (2015). Twist1 is a key regulator of Cancer-associated fibroblasts. Cancer Res.

[32] Spitzner M (2014). STAT3 inhibition sensitizes colorectal cancer to chemoradiotherapy in vitro and in vivo. Int J Cancer.

[33] Wörmann SM (2016). Loss of P53 Function Activates JAK2–STAT3 Signaling to Promote Pancreatic Tumor Growth, Stroma Modification, and Gemcitabine Resistance in Mice and Is Associated With Patient Survival. Gastroenterology.

[34] Meads MB, Gatenby RA, Dalton WS (2009). Environment-mediated drug resistance: a major contributor to minimal residual disease. Nat Rev Cancer.

[35] Ha SY (2014). The prognostic significance of cancer-associated fibroblasts in esophageal squamous cell carcinoma. PLoS One.

[36] Pavlides S (2009). The reverse Warburg effect: aerobic glycolysis in cancer associated fibroblasts and the tumor stroma. Cell Cycle.

[37] Ying H (2012). Oncogenic Kras maintains pancreatic tumors through regulation of anabolic glucose metabolism. Cell.

[38] Wang F-T (2019). Cancer-associated fibroblast regulation of tumor neo-angiogenesis as a therapeutic target in cancer. Oncol Lett.

[39] Provenzano PP (2012). Enzymatic targeting of the stroma ablates physical barriers to treatment of pancreatic ductal adenocarcinoma. Cancer Cell.

[40] Ohta M (2002). Monocyte chemoattractant protein-1 expression correlates with macrophage infiltration and tumor vascularity in human esophageal squamous cell carcinomas. Int J Cancer.

[41] Koide N (2004). Significance of macrophage chemoattractant protein-1 expression and macrophage infiltration in squamous cell carcinoma of the esophagus. Am J Gastroenterol.

[42] Shigeoka M (2013). Tumor associated macrophage expressing CD204 is associated with tumor aggressiveness of esophageal squamous cell carcinoma. Cancer Sci.

[43] Nabeki B (2015). Interleukin-32 expression and Treg infiltration in esophageal squamous cell carcinoma. Anticancer Res.

[44] Xia M (2013). Investigations on the clinical significance of FOXP3 protein expression in cervical oesophageal cancer and the number of FOXP3+ tumour-infiltrating lymphocytes. J Int Med Res.

[45] Osaki T (2009). Inverse correlation between NKG2D expression on CD8+ T cells and the frequency of CD4+CD25+ regulatory T cells in patients with esophageal cancer. Dis Esophagus.

[46] Xu T (2011). CD4 + CD25high regulatory T cell numbers and FOXP3 mRNA expression in patients with advanced esophageal cancer before and after chemotherapy. Cell Biochem Biophys.

[47] Bailey SR (2014). Th17 cells in cancer: the ultimate identity crisis. Front Immunol.

[48] Martin F, Apetoh L, Ghiringhelli F (2012). Controversies on the role of Th17 in cancer: a TGF-beta-dependent immunosuppressive activity?. Trends Mol Med.

[49] Chen D (2012). Increased IL-17-producing CD4(+) T cells in patients with esophageal cancer. Cell Immunol.

[50] Jiao ZJ (2012). Correlation between circulating myeloid-derived suppressor cells and Th17 cells in esophageal cancer. World J Gastroenterol.

[51] Nisar S (2020). Exploring Dysregulated signaling pathways in Cancer. Curr Pharm Des.

[52] Leu C-M (2003). Interleukin-6 acts as an antiapoptotic factor in human esophageal carcinoma cells through the activation of both STAT3 and mitogen-activated protein kinase pathways. Oncogene.

[53] Zhang Q (2015). STAT3 inhibitor stattic enhances radiosensitivity in esophageal squamous cell carcinoma. Tumor Biol.

[54] Yang L (2019). Novel activators and small-molecule inhibitors of STAT3 in cancer. Cytokine Growth Factor Rev.

[55] Lin L (2010). A novel small molecule inhibits STAT3 phosphorylation and DNA binding activity and exhibits potent growth suppressive activity in human cancer cells. Mol Cancer.

[56] Hatata T (2012). Immunohistochemical study of nuclear factor-κB expression in esophageal squamous cell carcinoma: prognostic significance and sensitivity to treatment with 5-FU. Dis Esophagus.

[57] Ogura M (2013). Clinical significance of CXCL-8/CXCR-2 network in esophageal squamous cell carcinoma. Surgery.

[58] Aldinucci D, Colombatti A (2014). The inflammatory chemokine CCL5 and cancer progression. Mediat Inflamm.

[59] Kameyoshi Y (1992). Cytokine RANTES released by thrombin-stimulated platelets is a potent attractant for human eosinophils. J Exp Med.

[60] Kuna P (1992). RANTES, a monocyte and T lymphocyte chemotactic cytokine releases histamine from human basophils. J Immunol.

[61] Mi S (2020). Radiotherapy increases 12-LOX and CCL5 levels in esophageal Cancer cells and promotes Cancer metastasis via THP-1-derived macrophages. Onco Targets Ther.

[62] Nie D (2003). Increased metastatic potential in human prostate carcinoma cells by overexpression of arachidonate 12-lipoxygenase. Clin Exp Metastasis.

[63] Wong BC (2001). 12-Lipoxygenase inhibition induced apoptosis in human gastric cancer cells. Carcinogenesis.

[64] Schneider C, Pozzi A (2011). Cyclooxygenases and lipoxygenases in cancer. Cancer Metastasis Rev.

[65] Jarnicki A, Putoczki T, Ernst M (2010). Stat3: linking inflammation to epithelial cancer - more than a "gut" feeling?. Cell Div.

[66] Akutsu Y (2011). COX2 expression predicts resistance to Chemoradiotherapy in esophageal squamous cell carcinoma. Ann Surg Oncol.

[67] Bielenberg DR, Zetter BR (2015). The Contribution of Angiogenesis to the Process of Metastasis. Cancer J (Sudbury, Mass).

[68] Xu M (2010). Regulation of antitumor immune responses by the IL-12 family cytokines, IL-12, IL-23, and IL-27. Clin Dev Immunol.

[69] Luo Y, Zheng SG. Hall of Fame among Pro-inflammatory Cytokines: Interleukin-6 Gene and Its Transcriptional Regulation Mechanisms. Front Immunol. 2016;7(604). 10.3389/fimmu.2016.00604 PMC5165036 28066415

[70] Chen M-F (2012). Role of interleukin 1 beta in esophageal squamous cell carcinoma. J Mol Med.

[71] Łukaszewicz-Zając M (2012). Higher importance of interleukin 6 than classic tumor markers (carcinoembryonic antigen and squamous cell cancer antigen) in the diagnosis of esophageal cancer patients. Dis Esophagus.

[72] Yousif NG (2013). Expression of IL-32 modulates NF-κB and p38 MAP kinase pathways in human esophageal cancer. Cytokine.

[73] Voronov E, Carmi Y, Apte RN (2014). The role IL-1 in tumor-mediated angiogenesis. Front Physiol.

[74] Chen S (2019). IL-1RA suppresses esophageal cancer cell growth by blocking IL-1α. J Clin Lab Anal.

[75] Zhou J (2018). IL-1beta from M2 macrophages promotes migration and invasion of ESCC cells enhancing epithelial-mesenchymal transition and activating NF-kappaB signaling pathway. J Cell Biochem.

[76] Yang S-m (2019). IL-11 activated by lnc-ATB promotes cell proliferation and invasion in esophageal squamous cell cancer. Biomed Pharmacother.

[77] Yue Y (2020). Interleukin-33-nuclear factor-κB-CCL2 signaling pathway promotes progression of esophageal squamous cell carcinoma by directing regulatory T cells. Cancer Sci.

[78] Hsing C-H (2013). Inhibiting interleukin-19 activity ameliorates esophageal squamous cell carcinoma progression. PLoS One.

[79] Martins-Green M, Petreaca M, Wang L (2013). Chemokines and their receptors are key players in the orchestra that regulates wound healing. Adv Wound Care.

[80] Miller MC, Mayo KH (2017). Chemokines from a structural perspective. Int J Mol Sci.

[81] Zlotnik A, Burkhardt AM, Homey B (2011). Homeostatic chemokine receptors and organ-specific metastasis. Nat Rev Immunol.

[82] Zlotnik A, Yoshie O (2012). The chemokine superfamily revisited. Immunity.

[83] Mehrad B, Keane MP, Strieter RM (2007). Chemokines as mediators of angiogenesis. Thromb Haemost.

[84] Goto M, Liu M (2020). Chemokines and their receptors as biomarkers in esophageal cancer. Esophagus.

[85] Łukaszewicz-Zając M (2016). The serum concentrations of chemokine CXCL12 and its specific receptor CXCR4 in patients with esophageal Cancer. Dis Markers.

[86] Fang H-Y (2018). CXCR4 Is a Potential Target for Diagnostic PET/CT Imaging in Barrett’s Dysplasia and Esophageal Adenocarcinoma. Clin Cancer Res.

[87] Tachezy M (2013). CXCR7 expression in esophageal cancer. J Transl Med.

[88] Wu K (2016). Silencing of CXCR2 and CXCR7 protects against esophageal cancer. Am J Transl Res.

[89] Ha H, Debnath B, Neamati N (2017). Role of the CXCL8-CXCR1/2 Axis in Cancer and inflammatory diseases. Theranostics.

[90] Łukaszewicz-Zając M (2019). Comparison between clinical significance of serum CXCL-8 and classical tumor markers in oesophageal cancer (OC) patients. Clin Exp Med.

[91] Liu J (2015). Local production of the chemokines CCL5 and CXCL10 attracts CD8+ T lymphocytes into esophageal squamous cell carcinoma. Oncotarget.

[92] Sato Y (2016). CXCL10 expression status is prognostic in patients with advanced thoracic esophageal squamous cell carcinoma. Ann Surg Oncol.

[93] Zhang H (2017). CAF-secreted CXCL1 conferred radioresistance by regulating DNA damage response in a ROS-dependent manner in esophageal squamous cell carcinoma. Cell Death Dis.

[94] Schaller TH (2017). Chemokines as adjuvants for immunotherapy: implications for immune activation with CCL3. Expert Rev Clin Immunol.

[95] Wu Y-C (2018). Autocrine CCL5 promotes tumor progression in esophageal squamous cell carcinoma in vitro. Cytokine.

[96] Liu J (2017). Expression of CCR6 in esophageal squamous cell carcinoma and its effects on epithelial-to-mesenchymal transition. Oncotarget.

[97] Shi M (2015). CCL21-CCR7 promotes the lymph node metastasis of esophageal squamous cell carcinoma by up-regulating MUC1. J Exp Clin Cancer Res.

[98] Ding Y (2003). Association of CC chemokine receptor 7 with lymph node metastasis of esophageal squamous cell carcinoma. Clin Cancer Res.

[99] Irino T (2014). CC-chemokine receptor CCR7: a key molecule for lymph node metastasis in esophageal squamous cell carcinoma. BMC Cancer.

[100] Hay ED (2005). The mesenchymal cell, its role in the embryo, and the remarkable signaling mechanisms that create it. Dev Dyn.

[101] Thuault S (2006). Transforming growth factor-beta employs HMGA2 to elicit epithelial-mesenchymal transition. J Cell Biol.

[102] Loh C-Y (2019). The E-cadherin and N-cadherin switch in epithelial-to-Mesenchymal transition: signaling, therapeutic implications, and challenges. Cells.

[103] Thiery JP (2002). Epithelial-mesenchymal transitions in tumour progression. Nat Rev Cancer.

[104] Davis FM (2014). Targeting EMT in cancer: opportunities for pharmacological intervention. Trends Pharmacol Sci.

[105] Gloushankova NA, Rubtsova SN, Zhitnyak IY (2017). Cadherin-mediated cell-cell interactions in normal and cancer cells. Tissue Barriers.

[106] Zhou J (2018). MCP2 activates NF-κB signaling pathway promoting the migration and invasion of ESCC cells. Cell Biol Int.

[107] Qiao Y (2018). IL6 derived from cancer-associated fibroblasts promotes chemoresistance via CXCR7 in esophageal squamous cell carcinoma. Oncogene.

[108] Goto M (2017). CXCR4 expression is associated with poor prognosis in patients with esophageal squamous cell carcinoma. Ann Surg Oncol.

[109] Zhang L (2013). The expressions of MIF and CXCR4 protein in tumor microenvironment are adverse prognostic factors in patients with esophageal squamous cell carcinoma. J Transl Med.

[110] Sullivan NJ (2009). Interleukin-6 induces an epithelial-mesenchymal transition phenotype in human breast cancer cells. Oncogene.

[111] Yadav A (2011). IL-6 promotes head and neck tumor metastasis by inducing epithelial-mesenchymal transition via the JAK-STAT3-SNAIL signaling pathway. Mol Cancer Res.

[112] Xiong H (2012). Roles of STAT3 and ZEB1 proteins in E-cadherin down-regulation and human colorectal cancer epithelial-mesenchymal transition. J Biol Chem.

[113] Gong C, et al. Abnormally expressed JunB transactivated by IL-6/STAT3 signaling promotes uveal melanoma aggressiveness via epithelial-mesenchymal transition. Biosci Rep. 2018;38(4). 10.1042/BSR20180532 PMC6028753 29899166

[114] Zang C (2017). IL-6/STAT3/TWIST inhibition reverses ionizing radiation-induced EMT and radioresistance in esophageal squamous carcinoma. Oncotarget.

[115] Chen M-F (2013). IL-6 expression predicts treatment response and outcome in squamous cell carcinoma of the esophagus. Mol Cancer.

[116] Ebbing EA (2019). Stromal-derived interleukin 6 drives epithelial-to-mesenchymal transition and therapy resistance in esophageal adenocarcinoma. Proc Natl Acad Sci U S A.

[117] Khan MA (2013). Twist: a molecular target in cancer therapeutics. Tumour Biol.

[118] Chen MF (2018). Role of ALDH1 in the prognosis of esophageal cancer and its relationship with tumor microenvironment. Mol Carcinog.

[119] Qian X (2016). Esophageal cancer stem cells and implications for future therapeutics. OncoTargets Ther.

[120] Chen D (2015). Interleukin-23 promotes the epithelial-mesenchymal transition of oesophageal carcinoma cells via the Wnt/beta-catenin pathway. Sci Rep.

[121] Bracken CP (2008). A double-negative feedback loop between ZEB1-SIP1 and the microRNA-200 family regulates epithelial-mesenchymal transition. Cancer Res.

[122] Korpal M (2008). The miR-200 family inhibits epithelial-mesenchymal transition and cancer cell migration by direct targeting of E-cadherin transcriptional repressors ZEB1 and ZEB2. J Biol Chem.

[123] Park SM (2008). The miR-200 family determines the epithelial phenotype of cancer cells by targeting the E-cadherin repressors ZEB1 and ZEB2. Genes Dev.

[124] Burk U (2008). A reciprocal repression between ZEB1 and members of the miR-200 family promotes EMT and invasion in cancer cells. EMBO Rep.

[125] Wu BL (2011). MiRNA profile in esophageal squamous cell carcinoma: downregulation of miR-143 and miR-145. World J Gastroenterol.

[126] Zhang HF (2014). miR-200b suppresses invasiveness and modulates the cytoskeletal and adhesive machinery in esophageal squamous cell carcinoma cells via targeting Kindlin-2. Carcinogenesis.

[127] Yang N (2018). MicroRNAs: pleiotropic regulators in the tumor microenvironment. Front Immunol.

[128] Yang J (2014). MicroRNA-19a-3p inhibits breast cancer progression and metastasis by inducing macrophage polarization through downregulated expression of Fra-1 proto-oncogene. Oncogene.

[129] Bullock MD (2013). Pleiotropic actions of miR-21 highlight the critical role of deregulated stromal microRNAs during colorectal cancer progression. Cell Death Dis.

[130] Naito Y (2014). MicroRNA-143 regulates collagen type III expression in stromal fibroblasts of scirrhous type gastric cancer. Cancer Sci.

[131] Mei S (2015). MicroRNA-200c promotes suppressive potential of myeloid-derived suppressor cells by modulating PTEN and FOG2 expression. PLoS One.

[132] Chakraborty C (2020). The interplay among miRNAs, major cytokines, and Cancer-related inflammation. Mol Ther Nucleic Acids.

[133] Zhang M (2013). Role of cancer-related inflammation in esophageal cancer. Crit Rev Eukaryot Gene Expr.

[134] Zhang M (2017). miR-302b inhibits cancer-related inflammation by targeting ERBB4, IRF2 and CXCR4 in esophageal cancer. Oncotarget.

[135] Cheng Y (2015). STAT3 is involved in miR-124-mediated suppressive effects on esophageal cancer cells. BMC Cancer.

[136] Zhang M (2014). miR-302b is a potential molecular marker of esophageal squamous cell carcinoma and functions as a tumor suppressor by targeting ErbB4. J Exp Clin Cancer Res.

[137] Fares J (2020). Molecular principles of metastasis: a hallmark of cancer revisited. Signal Transduct Target Ther.

[138] Joyce JA, Pollard JW (2009). Microenvironmental regulation of metastasis. Nat Rev Cancer.

[139] Vandercappellen J, Van Damme J, Struyf S (2008). The role of CXC chemokines and their receptors in cancer. Cancer Lett.

[140] Zhao H (2015). CXCR4 over-expression and survival in cancer: a system review and meta-analysis. Oncotarget.

[141] Lu CL (2014). Cxcr4 heterogeneous expression in esophageal squamous cell cancer and stronger metastatic potential with Cxcr4-positive cancer cells. Dis Esophagus.

[142] Ben-Baruch A (2009). Site-specific metastasis formation: chemokines as regulators of tumor cell adhesion, motility and invasion. Cell Adhes Migr.

[143] Kaifi JT (2005). Tumor-cell homing to lymph nodes and bone marrow and CXCR4 expression in esophageal Cancer. J Natl Cancer Institute.

[144] Koishi K (2006). Persistent CXCR4 expression after preoperative chemoradiotherapy predicts early recurrence and poor prognosis in esophageal cancer. World J Gastroenterol.

[145] Sasaki K (2009). Expression of CXCL12 and its receptor CXCR4 in esophageal squamous cell carcinoma. Oncol Rep.

[146] Gros SJ (2012). CXCR4/SDF-1alpha-mediated chemotaxis in an in vivo model of metastatic esophageal carcinoma. In Vivo.

[147] Wang X (2017). Stem cell autocrine CXCL12/CXCR4 stimulates invasion and metastasis of esophageal cancer. Oncotarget.

[148] Lu C (2015). Clinical and biological significance of stem-like CD133(+)CXCR4(+) cells in esophageal squamous cell carcinoma. J Thorac Cardiovasc Surg.

[149] Balkwill F (2004). Cancer and the chemokine network. Nat Rev Cancer.

[150] Ma H (2015). CCR7 enhances TGF-β1-induced epithelial-mesenchymal transition and is associated with lymph node metastasis and poor overall survival in gastric cancer. Oncotarget.

[151] Pang MF (2016). TGF-β1-induced EMT promotes targeted migration of breast cancer cells through the lymphatic system by the activation of CCR7/CCL21-mediated chemotaxis. Oncogene.

[152] Xiong Y (2017). CCL21/CCR7 interaction promotes cellular migration and invasion via modulation of the MEK/ERK1/2 signaling pathway and correlates with lymphatic metastatic spread and poor prognosis in urinary bladder cancer. Int J Oncol.

[153] Song Y (2012). CCR7 and VEGF-C: molecular Indicator of lymphatic metastatic recurrence in pN0 esophageal squamous cell carcinoma after Ivor-Lewis Esophagectomy?. Ann Surg Oncol.

[154] Kitadai Y (2001). Clinicopathological significance of vascular endothelial growth factor (VEGF)-C in human esophageal squamous cell carcinomas. Int J Cancer.

[155] Zhang W (2012). Expression and significance of vascular endothelial growth factor C from multiple specimen sources in esophageal squamous cell carcinoma. Int J Biol Markers.

[156] Issa A (2009). Vascular endothelial growth factor-C and C-C chemokine receptor 7 in tumor cell-lymphatic cross-talk promote invasive phenotype. Cancer Res.

[157] Shih C-H (2000). Vascular endothelial growth factor expression predicts outcome and lymph node metastasis in squamous cell carcinoma of the esophagus. Clin Cancer Res.

[158] Shimada H (2001). Clinical significance of serum vascular endothelial growth factor in esophageal squamous cell carcinoma. Cancer.

[159] Hirakawa S (2005). VEGF-A induces tumor and sentinel lymph node lymphangiogenesis and promotes lymphatic metastasis. J Exp Med.

[160] Hirakawa S (2007). VEGF-C-induced lymphangiogenesis in sentinel lymph nodes promotes tumor metastasis to distant sites. Blood.

[161] Garmy-Susini B (2013). PI3Kalpha activates integrin alpha4beta1 to establish a metastatic niche in lymph nodes. Proc Natl Acad Sci U S A.

[162] Liu Q (2016). The CXCL8-CXCR1/2 pathways in cancer. Cytokine Growth Factor Rev.

[163] Sui P (2013). High expression of CXCR-2 correlates with lymph node metastasis and predicts unfavorable prognosis in resected esophageal carcinoma. Med Oncol.

[164] Krzystek-Korpacka M (2008). Elevation of circulating interleukin-8 is related to lymph node and distant metastases in esophageal squamous cell carcinomas--implication for clinical evaluation of cancer patient. Cytokine.

[165] Hannelien V (2012). The role of CXC chemokines in the transition of chronic inflammation to esophageal and gastric cancer. Biochimica et Biophysica Acta (BBA) Rev Cancer.

[166] Hosono M (2017). CXCL8 derived from tumor-associated macrophages and esophageal squamous cell carcinomas contributes to tumor progression by promoting migration and invasion of cancer cells. Oncotarget.

[167] Waugh DJJ, Wilson C (2008). The Interleukin-8 pathway in Cancer. Clin Cancer Res.

[168] Dvorak HF (1986). Tumors: wounds that do not heal. Similarities between tumor stroma generation and wound healing. N Engl J Med.

[169] Berraondo P (2019). Cytokines in clinical cancer immunotherapy. Br J Cancer.

[170] Wagener-Ryczek S (2020). Immune profile and immunosurveillance in treatment-naive and neoadjuvantly treated esophageal adenocarcinoma. Cancer Immunol Immunother.

[171] Paul S, Lal G (2017). The molecular mechanism of natural killer cells function and its importance in Cancer immunotherapy. Front Immunol.

[172] Wu J (2019). IL-6 and IL-8 secreted by tumour cells impair the function of NK cells via the STAT3 pathway in oesophageal squamous cell carcinoma. J Exp Clin Cancer Res.

[173] Wculek SK (2020). Dendritic cells in cancer immunology and immunotherapy. Nat Rev Immunol.

[174] Shurin MR (2006). Intratumoral cytokines/chemokines/growth factors and tumor infiltrating dendritic cells: friends or enemies?. Cancer Metastasis Rev.

[175] Lin A (2010). Dendritic cells integrate signals from the tumor microenvironment to modulate immunity and tumor growth. Immunol Lett.

[176] Somja J (2013). Dendritic cells in Barrett's esophagus carcinogenesis: an inadequate microenvironment for antitumor immunity?. Am J Pathol.

[177] Ohue Y, Nishikawa H (2019). Regulatory T (Treg) cells in cancer: can Treg cells be a new therapeutic target?. Cancer Sci.

[178] Fridman WH, et al. The immune contexture in human tumours: impact on clinical outcome. Nat Rev Cancer. 2012:298–306. 10.1038/nrc3245 22419253

[179] Liu JY (2015). CTL- vs Treg lymphocyte-attracting chemokines, CCL4 and CCL20, are strong reciprocal predictive markers for survival of patients with oesophageal squamous cell carcinoma. Br J Cancer.

[180] Wang WL (2015). Quantification of tumor infiltrating Foxp3+ regulatory T cells enables the identification of high-risk patients for developing synchronous cancers over upper aerodigestive tract. Oral Oncol.

[181] Murai M (2009). Interleukin 10 acts on regulatory T cells to maintain expression of the transcription factor Foxp3 and suppressive function in mice with colitis. Nat Immunol.

[182] Smith AJ (2011). The immunosuppressive role of IL-32 in lymphatic tissue during HIV-1 Infection1. J Immunol.

[183] Lin Y, Xu J, Lan H (2019). Tumor-associated macrophages in tumor metastasis: biological roles and clinical therapeutic applications. J Hematol Oncol.

[184] Yang H, et al. CCL2-CCR2 axis recruits tumor associated macrophages to induce immune evasion through PD-1 signaling in esophageal carcinogenesis. Mol Cancer. 2020. 10.1186/s12943-020-01165-x PMC7045401 32103760

[185] Yagi T (2019). Tumour-associated macrophages are associated with poor prognosis and programmed death ligand 1 expression in oesophageal cancer. Eur J Cancer.

[186] Derks S (2015). Epithelial PD-L2 expression Marks Barrett's esophagus and esophageal adenocarcinoma. Cancer Immunol Res.

[187] Gao J (2014). Infiltration of alternatively activated macrophages in cancer tissue is associated with MDSC and Th2 polarization in patients with esophageal cancer. PLoS One.

[188] Kumar V (2016). The nature of myeloid-derived suppressor cells in the tumor microenvironment. Trends Immunol.

[189] Chen MF (2014). IL-6-stimulated CD11b+CD14+HLA-DR− myeloid-derived suppressor cells, are associated with progression and poor prognosis in squamous cell carcinoma of the esophagus. Oncotarget.

[190] Karakasheva TA (2015). CD38-expressing myeloid-derived suppressor cells promote tumor growth in a murine model of esophageal Cancer. Cancer Res.

[191] Chen X (2018). Dual TGF-beta and PD-1 blockade synergistically enhances MAGE-A3-specific CD8(+) T cell response in esophageal squamous cell carcinoma. Int J Cancer.

[192] Wang D-f (2013). Effects of CXCR4 gene silencing by lentivirus shRNA on proliferation of the EC9706 human esophageal carcinoma cell line. Tumor Biol.

[193] Lidonnici MR (2017). Plerixafor and G-CSF combination mobilizes hematopoietic stem and progenitors cells with a distinct transcriptional profile and a reduced in vivo homing capacity compared to plerixafor alone. Haematologica.

[194] Doi T (2018). Safety and antitumor activity of the anti-programmed Death-1 antibody Pembrolizumab in patients with advanced esophageal carcinoma. J Clin Oncol.

[195] Shah MA (2019). Efficacy and safety of Pembrolizumab for heavily pretreated patients with advanced, metastatic adenocarcinoma or squamous cell carcinoma of the esophagus: the phase 2 KEYNOTE-180 study. JAMA Oncol.

[196] Shah MA (2019). Pembrolizumab versus chemotherapy as second-line therapy for advanced esophageal cancer: Phase 3 KEYNOTE-181 study. J Clin Oncol.

[197] Enzinger PC (2018). Multicenter phase II trial of pembrolizumab (pembro) in previously-treated metastatic esophageal cancer. J Clin Oncol.

[198] Kudo T (2017). Nivolumab treatment for oesophageal squamous-cell carcinoma: an open-label, multicentre, phase 2 trial. Lancet Oncol.

[199] Janjigian YY (2018). CheckMate-032 study: efficacy and safety of Nivolumab and Nivolumab plus Ipilimumab in patients with metastatic Esophagogastric Cancer. J Clin Oncol.

[200] Janjigian YY (2019). First-line pembrolizumab (P), trastuzumab (T), capecitabine (C) and oxaliplatin (O) in HER2-positive metastatic esophagogastric adenocarcinoma (mEGA). J Clin Oncol.

[201] Stroes CI (2020). Phase II feasibility and biomarker study of Neoadjuvant Trastuzumab and Pertuzumab with Chemoradiotherapy for Resectable human epidermal growth factor receptor 2-positive esophageal adenocarcinoma: TRAP study. J Clin Oncol.

[202] Dutton SJ (2014). Gefitinib for oesophageal cancer progressing after chemotherapy (COG): a phase 3, multicentre, double-blind, placebo-controlled randomised trial. Lancet Oncol.

[203] Zhang X (2019). Nimotuzumab plus paclitaxel and Cisplatin as a 1(st)-line treatment for esophageal Cancer: long term follow-up of a phase II study. J Cancer.

[204] Yasuda T (2017). Cancer peptide vaccine to suppress postoperative recurrence in esophageal SCC patients with induction of antigen-specific CD8+ T cell. J Clin Oncol.

[205] Kono K (2009). Vaccination with multiple peptides derived from novel cancer-testis antigens can induce specific T-cell responses and clinical responses in advanced esophageal cancer. Cancer Sci.

[206] Fujiwara T (2019). Abstract CT185: phase I dose-escalation study of endoscopic intratumoral injection of OBP-301 (telomelysin) with radiotherapy in esophageal cancer patients unfit for standard treatments. Cancer Res.

[207] Fuchs CS (2014). Ramucirumab monotherapy for previously treated advanced gastric or gastro-oesophageal junction adenocarcinoma (REGARD): an international, randomised, multicentre, placebo-controlled, phase 3 trial. Lancet.

[208] Wilke H (2014). Ramucirumab plus paclitaxel versus placebo plus paclitaxel in patients with previously treated advanced gastric or gastro-oesophageal junction adenocarcinoma (RAINBOW): a double-blind, randomised phase 3 trial. Lancet Oncol.

[209] Pant S (2017). A phase II study of the c-met inhibitor Tivantinib in combination with FOLFOX for the treatment of patients with previously untreated metastatic adenocarcinoma of the distal esophagus, Gastroesophageal junction, or stomach. Cancer Investig.

[210] Sahin U (2018). A phase I dose-escalation study of IMAB362 (Zolbetuximab) in patients with advanced gastric and gastro-oesophageal junction cancer. Eur J Cancer.

[211] Schuler MH (2017). Expression of Claudin 18.2 and HER2 in gastric, gastroesophageal junction, and esophageal cancers: Results from the FAST study. J Clin Oncol.

[212] Bendell JC (2016). Phase I study of DKN-01, an anti-DKK1 antibody, in combination with paclitaxel (pac) in patients (pts) with DKK1+ relapsed or refractory esophageal cancer (EC) or gastro-esophageal junction tumors (GEJ). J Clin Oncol.

[213] Smyth EC (2016). Phase II study of AZD4547 in FGFR amplified tumours: Gastroesophageal cancer (GC) cohort pharmacodynamic and biomarker results. J Clin Oncol.

[214] Iinuma H (2014). Phase I clinical study of multiple epitope peptide vaccine combined with chemoradiation therapy in esophageal cancer patients. J Transl Med.

[215] Chao J (2016). Pilot trial of CRLX101 in patients (pts) with advanced, chemotherapy-refractory gastroesophageal cancer (GEC). J Clin Oncol.

[216] Shrivastava MS (2014). Targeting chemokine pathways in esophageal adenocarcinoma. Cell cycle (Georgetown, Tex).

[217] Li Y (2017). Immune signature profiling identified predictive and prognostic factors for esophageal squamous cell carcinoma. Oncoimmunology.

